# Does the U.S. Navy’s reliance on objective standards prevent discrimination in promotions and retentions?

**DOI:** 10.1371/journal.pone.0250630

**Published:** 2021-04-28

**Authors:** Amos Golan, William H. Greene, Jeffrey M. Perloff

**Affiliations:** 1 Department of Economics, American University, Washington, D.C., United States of America; 2 Santa Fe Institute, Santa Fe, NM, United States of America; 3 Stern School of Business, New York University, New York, NY, United States of America; 4 Department of Agricultural and Resource Economics, University of California, Berkeley, CA, United States of America; Iowa State University, UNITED STATES

## Abstract

To prevent discrimination, the U.S. Navy enlisted-personnel promotion process relies primarily on objective measures. However, it also uses the subjective opinion of a sailor’s superior. The Navy’s promotion and retention process involves two successive decisions: The Navy decides whether to promote an individual, and conditional on that decision, the sailor decides whether to stay. Using estimates of these correlated decision-making processes, we find that during 1997–2008, Blacks and Hispanics were less likely to be promoted than Whites, especially during wartime. The Navy’s decision-making affects Blacks’ differential promotion rates by twice as much as differences in the groups’ characteristics. However, Nonwhite retention probabilities, even when not promoted, are higher than for Whites, in part because they have fewer opportunities in the civilian market. Females have lower promotion rates than males and slightly lower retention rates during wartime.

## Introduction

Can the U.S. Navy prevent the race- and sex-based glass ceilings that are rife in civilian labor markets? To combat such discrimination, the U.S. Navy has instituted formal job evaluations for its enlisted personnel [1]. In its promotion process, four of the five factors that determine promotion are reasonably objective performance measures. However, one factor is subjective: an evaluation by the sailor’s superior. Does this one subjective factor result in promotion rates that vary by race, ethnicity, or sex? If the Navy’s formal, primarily objective, system works, it can provide a model for other employers. If it fails, then the Navy needs new measures to prevent unequal treatment of minorities and women.

The Navy believes that using objective standards is vital to preventing discrimination and unequal treatment in promotions. Minorities and males constitute a larger share of the Navy than of the civilian population. During the late 1990’s through about 2010 about 40% of the enlisted sailors (pay grade E1 –E6) were Nonwhite, a bit more than a fifth were Black, and about 16% were female. In 2020, for example, about half of enlisted sailors (pay grades E1–E6) were Nonwhite, nearly a fifth are Black, and about 16% are female [2, 3].

**Fig 1:**
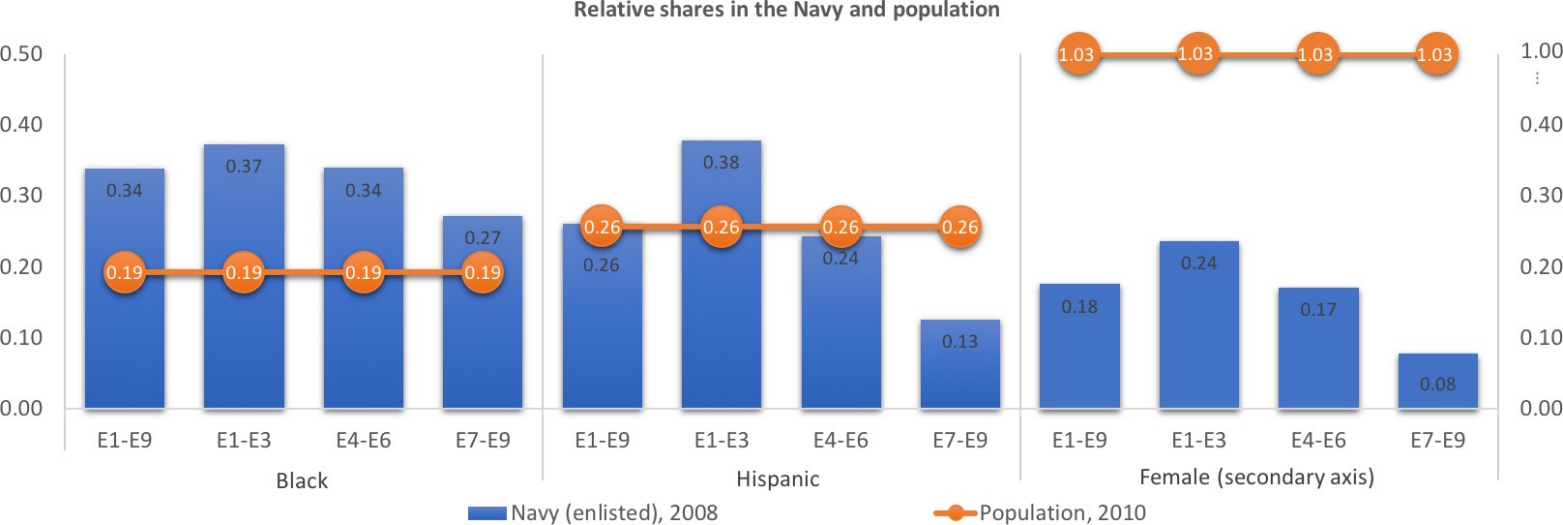
The relative frequencies of minorities in the population (2010 census) and in the Navy (2008).

Fig 1 shows the relative frequencies of minorities in the United States population (census 2010) and in the Navy at the end of the period we study (2008). The “relative frequencies” are the proportion of a subgroup (say, Blacks) divided by that of Whites, or the female frequency divided by the male frequency. The orange values show the population, while the blue ones show the Navy’s relative frequencies. The proportions of Blacks in all Navy paygrades are much higher than their overall population proportion. The proportions of Hispanics are similar (or lower) than Whites except for paygrade E1-E3. The proportions of females are much lower than that of males in all paygrades.

Retention rates depend on the sailors’ opportunities in the civilian market and promotions within the Navy, as well as on an individual’s willingness to serve the country. The Navy has worked hard to increase its retention rates to maintain national security. Because a sailor’s reenlistment decision is affected by the Navy’s promotion decision, unequal promotion rates across demographic groups may affect who stays in the Navy.

To investigate whether promotion and retention rates vary by race, ethnicity, and gender, we present the first consistently estimated promotion and retention model. Virtually all previous studies of military and civilian labor forces look at only one or the other of these decisions, look at the two decisions separately, or do not consistently estimate these sequential decisions.

We use a two-step decision model. In the first stage, the Navy decides whether to promote sailors based on their current and past performances and the Navy’s current needs. Second, sailors decide whether to remain in the Navy or leave, conditional on whether they receive a promotion and other Navy decisions such as whether they are assigned sea duty. Promotion decisions and decisions whether a sailor stays depend on Navy policies (including demand and supply), individuals’ characteristics, economic conditions in the civilian labor market, and conditions of war or peace. Our analysis uses data covering the universe of Navy enlisted personnel from January 1997 through May 2008. Thus, it covers both peacetime before the 9/11 attacks and subsequently wartime, with both periods of roughly the same length.

Consequently, we can investigate whether a fundamental change in promotion and retention rates occurred following 9/11. Such a change could be due to an increased need for experienced sailors during wartime, individuals’ greater willingness to serve their nation, or another change in the economic environment.

Two of the earliest and best-known promotion studies are [4, 5]. The authors investigated the relationship between personal attributes and job performance as measured by the rate of promotion. The basic model is a degenerate (most states are zero) first-order Markov transition model. The firm decides whether to promote, and the individual decides whether to stay or leave, but these decisions are not estimated separately. The transition probability is estimated independently of the grade level (rank) using a maximum likelihood method.

Other methods used to study promotions include ordered multinomial models [6], multinomial systems of censored equations [7], random-effects models [8], and Markov promotion models [9]. There are surprisingly few recent papers on promotion and retention in civilian labor markets. The few that exist do not simultaneously examine promotion and retention, such as the study of baseball by [10].

Our superior data allow us to use a methodology that differs from previous studies in two critical ways. First, we control for individuals’ abilities much more thoroughly than in studies of civilian employers. We have extensive data on individuals’ current and past performance and their aptitude and ability as measured by the individual’s Armed Forces Qualifying Test (AFQT) score. The AFQT is given to all recruits in the U.S. armed forces and measures their arithmetic reasoning, mathematics knowledge, paragraph comprehension, and word knowledge [11]. Unlike other studies, we have also linked individual-specific civilian labor market data to capture sailors’ options in the stay-or-leave decision. We measure their options using their expected wage and employment probability conditional on their skills, demographics, and the state of the economy.

Second, we simultaneously estimate equations for the Navy’s promotion decision and the individual’s retention decision. Most previous studies, particularly Navy studies, examine only whether an individual was both promoted and stayed with the firm. Those few studies that address promotion and retention separately do not formally analyze the link between these decisions.

While many articles have been written on wage and other forms of discrimination [12, 13], relatively few articles address the role of discrimination in promotion and retention, particularly in the Navy. Several analyses—most of which are primarily descriptive—concluded that promotion rates in the Navy and other armed forces vary by race or gender. These studies examined the combined outcome that one is promoted and stayed in the armed force. For example, [14, 15] showed that such promotion rates differ by race in the U.S. military, and [16] presented similar results for the Royal Navy based on class. In the probit model in [17], a variable is one if a Marine Corps officer is promoted and remains in the Corps. Other studies such as [18, 19] looked at retention in the military but did not examine the promotion decision. Some Center for Naval Analyses studies [20–22] examined various aspects of officer career progressions.

A few studies addressed, at least descriptively, both promotion and retention. A gender differences in promotion and retention rates in the U.S. Air Force was found in [23]. One study [24] observed a link between the rate of promotion and retention of enlisted personnel. Another study [25] looked at civil service workers in the Department of Defense. It estimated separate promotion and separation regressions using survival analysis (Cox proportional-hazard model).

The study that is most similar to our work is [26]. It examined the relationship between recruit quality and promotion speed of the U.S. Navy’s first-term enlisted personnel and the effect on attrition using a two-stage model. The first stage models the time until promotion (which implicitly assumes that the sailor remains within the Navy). The second stage is a probit model for the probability of attrition that includes the predicted hazard rate of promotion. Consequently, even if these estimates were consistent, they would not allow one to determine how the promotion rate affects the probability that a sailor remains in the Navy, unlike in our study.

## Institutional promotion rules

The Navy imposes many requirements on its promotion process. One of its chief motivations for its formal process is to prevent discrimination.

Enlisted sailors are assigned to pay grades E1 through E9. A sailor must spend a minimum time in any given grade before promotion is possible, with few exceptions. After sailors have spent the minimal period in a grade and demonstrated a minimal level of performance, their probability of promotion is positive until they have spent the maximum permitted time in that grade without a promotion and must leave the Navy.

Sailors start in pay grade E1 and receive virtually automatic promotions to E2 and then E3. Promotions to higher ranks are not guaranteed. The promotions we examine, from E4 through E6, are based on primarily objective performance evaluations. (Our model also captures demotions: moving to a lower pay grade. We do not discuss demotions because the data contain only a handful of such cases.) Promotion to E7 adds a record review by a selection board. A selection board decides whether to promote someone to E8 or E9 based on several factors, some of which are highly subjective. Consequently, we concentrate on promotions to E4 through E7, where fewer factors are subjective.

The Navy bases promotions to ranks E4 through E7 on an individual’s Final Multiple score, a pay grade-specific value for each individual in each promotion period. The Navy promotes sailors, within skill groups and specialties, to the next pay grade starting with the highest individual Final Multiple score until it fills all its vacancies in that pay grade. The Final Multiple score for promotions to Grades E4 through E6 is a weighted average of five components: time in grade, the Performance Mark Average (PMA), an examination score, the Pass Not Advance (PNA) measure, and awards. Most of the aspects of this process are objective and leave no room for discrimination.

The first component of the Final Multiple score for ranks E4 through E6 is time in service in a pay grade. Individuals cannot take the required pre-promotion exam until they meet the minimum time in grade (TIR), which varies by pay grade.

The second component is the Performance Mark Average (PMA). The PMA uses the individual’s fitness report, FITREP. A supervisor evaluates an individual on teamwork, leadership, and other factors. Each sailor is assigned a PMA (four-point scale) score. An individual must receive a PMA greater than or equal to 3.6 to be eligible to take the pre-promotion exam: Early Promote (4.0 points), Must Promote (3.8), Promotable (3.6), Progressing (3.4), and Significant Problems (2.0 points). The Navy has tried to force performance mark averages into a bell curve across all individuals with limited success. If discrimination or other non-objective criteria enter the Final Multiple score, they enter through the PMA.

The third component is the pre-promotion examination score. All eligible individuals must take the exam. In each promotion cycle (approximately every six months depending on the pay grade), the Navy sets a cut score: the minimum exam score for an individual to be eligible for a promotion. An individual who fails to pass must take another exam in the next promotion cycle. In our empirical work, we use a variable called Pass, which is one if the sailor takes and passes this test and the Final Multiple exceeds the cut score.

The fourth factor is the Pass Not Advance (PNA) measure. Individuals who did not receive a promotion the first time they were eligible because of a lack of vacant positions are awarded PNA points. These points increase the individuals’ Final Multiple value in the next evaluation period, giving these individuals a slight advantage over first-time test takers.

The fifth component is Awards. There are 28 different awards listed for which points can be earned, with points varying across these awards. For example, a sailor receives 10 points for the Medal of Honor, 5 points for the Navy Cross, and 2 points for an Executive Letter of Commendation. Such major awards are extremely uncommon.

Thus, the Final Multiple is a function of four objective measures and one subjective measure, the PMA. More specifically, for promotions to Grades E4 and E5, Multiple = 0.34×Score + 0.36[(PMA×60)– 156] + 0.13[(TIR×2) + 15] + 0.13(PNA×2), where Score is the promotion test score. For Grade E6, Multiple = 0.30×Score + 0.415[(PMA×60)– 130] + 0.13[(TIR×2) + 19] + 0.11(PNA× 2). For Grade E7, Multiple = 0.60×Score + 0.40(PMA×13).

Some subjectivity may also enter into decisions to promote a few people outside of this system—early promotion—or decide which sailor among several with the same Final Multiple score receives a promotion. Finally, promotion from E6 to E7 involves one more stage that introduces subjectivity. An advancement board similar to the promotion boards for officers considers all candidates with adequate final multiples. The panel of raters compares candidates against each other. The panel uses an iterative process to reach some pre-established number of advancements.

Regardless of whether they receive a promotion, sailors must decide whether to re-enlist for an additional period toward the end of their current contract. Most sailors who re-enlist do so for four years, though they may also be able to re-enlist for five or six years. Under certain circumstances, sailors can extend their service for up to two years [27, 28].

All sailors are subject to the High Tenure Policy, which sets a maximum time by which an individual must receive a promotion to the next pay grade from the current one. However, we did not observe any cases that used the High Tenure Policy. Individuals unlikely to get a promotion receive a signal and leave well before they hit their maximum time limit. Consistent with this observation, we did not observe changes in average tenures when the rules on High Tenure changed.

## Data

The Navy data set covers all enlisted sailors in every skill group (occupation) and pay grade (E3 through E7) from January 1997 through May 2008. We drop one-seventh of the observations due to missing data for some variables, which does not change the moments of the original data set. Thus, the missing values are random.

We start following sailors in the month when they first received a promotion from January 1997 through May 2008. We then record information about that individual for each successive 12-month period. (We experimented with shorter and longer periods and found that our results are insensitive to the length of this interval.) We follow sailors until they exit the sample, or we reach the final period. We have no natural birth cohort or period cohort. However, we observe a promotion cohort: people eligible for promotion at each promotion cycle at each pay grade and occupation.

We consider all completed promotion decisions within our time frame, dropping the remaining data at the end of our sample period. Consequently, the data set may be truncated from the left—if the first promotion occurred in a year before the first time we observe an individual in the data—or from the right—if the last observed promotion occurred after the last period, May 2008. Truncation does not create a problem because we have all the completed promotion/stay decisions during the relevant period.

Because an individual’s first observation in our data set is associated with a promotion decision, we do not use information about prior promotion decisions. Using this approach, we can measure the length of time from the last promotion to a given promotion decision. The S1 Appendix provide a complete description of our data.

## Model

To examine the joint decisions of the Navy whether to promote a sailor and the individual’s decision whether to remain in the Navy, we estimate a recursive bivariate probit model. In the first equation, the Navy decides whether to promote based on its assessment of an individual’s performance and ability and its need for sailors. In the second equation, the individual’s decision to remain in the Navy or leave is conditional on whether the individual receives a promotion. (In some cases, the sailor is already in the middle of an enlistment period, so they cannot leave immediately, but we know if they left in the following period.) We proxy the individuals’ preferences and outside options by demographic variables and civilian economic conditions. The Navy’s and an individual’s decisions are correlated.

Let *y*_*i*1_ be a binary variable that equals one if the Navy promotes an individual (and zero otherwise), and *y*_*i*2_ is a binary variable that equals one if the sailor remains in the Navy following the promotion decision. We estimate a Maximum Likelihood (ML) probit system that reflects the endogeneity of the promotion offer in the retention decision [29] where sailor *i*’s decision, *y*_*i*2_, is conditional on the Navy’s decision, *y*_*i*1_, about sailor *i*. Thus,

$$\begin{array}{l} z_{i1}=\beta_{1}^{'}x_{i1}+\varepsilon_{i1},\;y_{i1}=\mathrm{sign}\left(z_{i1}\right)\\ z_{i2}=\beta_{2}^{'}x_{i2}+\gamma y_{i1}+\varepsilon_{i2},\;y_{i2}=\mathrm{sign}\left(z_{i2}\right) \end{array}$$

where *z*_*i*1_ is the latent variable related to whether the Navy promotes the individual, and *z*_*i*2_ is the latent variable for reenlistment. We assume that the random elements follow a bivariate normal distribution [*ε*_*i*1_, *ε*_*i*2_]~BVN[0,0,1,1,*ρ*] where ρ is the correlation coefficient between the two equations [30–32]. Given the large sample size, the normality assumption is reasonable.

We estimate these probabilities and ρ in a bivariate probit model using maximum likelihood (ML). The ML estimator accounts for the endogeneity of *y*_*i*1_ in the second equation. In a linear system, one uses instrumental variables to address endogeneity. In our nonlinear system, the explanatory variables in the Navy’s decision that do not appear in the sailor’s retention decision, such as the Navy’s staffing needs, serve the function of instruments indirectly through their determination of *y*_*i*1_.

The two equations contain some common demographic variables. Variables in both equations include: a sailor’s Armed Forces Qualifications Test (AFQT) percentile score; education variables; whether a sailor is currently on sea duty, a separate dummy variable for each current pay grade, E4, E5, and E6; race-ethnicity and sex dummies; and time dummies. (The Navy accepted only individuals with AFQT percentile scores above 30. This criterion rose to 35 in July 2003).

For most of the period analyzed, the Navy’s classification scheme treated Hispanic as a race rather than as an ethnicity so that no sailor was classified as both White and Hispanic or Black and Hispanic. Consequently, we look at four racial-ethnic groups: White, Black, Hispanic, and Other (primarily Asian-Americans). We use White as the base group, as it is the largest group. We interact the pay grade dummies with the race-ethnicity and female dummies to test for demographic group differences in promotion and retention across the pay grades.

Our data period is nearly evenly divided into peacetime before September 11, 2011, and wartime afterward. Because the Navy’s need for experienced sailors may have changed during wartime and civilian opportunities changed, we allow for the possibility that promotion and retention equations shifted when the country went to war (a possible structural change). We use five time variables. A September 11, 2001 dummy captures a once-and-for-all shift from the “peace” to the “war” period. The Peacetime variable is a time trend that increases by one each year through September 2001, and then is zero. Wartime is a time trend that is zero before September 2001 and then increases by one each year. We also include squared terms for both these time trends. The trend variables capture changes in the ratio of the civilian wage to the Navy wage.

The remaining variables appear in only one of the equations. The variables that are only in the promotion equation are the Navy policies we discussed earlier. These variables serve as instruments for the retention equation. To capture possible Navy policy changes not otherwise captured by explicit variables or changes in supervisors’ attitudes over time that might affect the probability of promotion, we interact the race and sex dummies with the time trends. (Although we know of no reason why these interaction variables should appear in the retention equation, we experimented with including them. However, all of their t-statistics were virtually zero).

The military designed the AFQT to be an unbiased test across demographic groups [33]. However, because it tests school-taught mathematical and verbal skills, if some demographic groups receive inferior educations, their AFQT scores could be systematically lower than those of other groups. To capture any such effect, we interact the AFQT score with the demographic group dummies.

In addition to the dummy for current assignment to sea duty, the promotion equation includes two sea duty measures over a sailor’s career to date: the share of time in the Navy spent on sea duty and the share squared. We excluded these variables from the retention equation because the share of one’s career already spent at sea is a “sunk cost”.

The Pass dummy shows whether an individual was eligible to take the exam and passed it. It is not a perfect indicator because the Navy occasionally promotes people who have not taken or passed the exam if the Navy has an extraordinary need for personnel in certain positions.

The Navy chooses whether to promote all eligible sailors depending on demand and supply conditions. The Navy’s promotion demand variable is Vacancies, the number of open positions to be filled by promotions for an exam cycle (time period) for a given pay grade and occupation. Takers is the number of sailors who are potentially eligible for promotion, at the same period, pay grade, skill, and specialty, and have taken and passed the exam. The promotion equation includes the ratio of demand to supply (a measure of the excess supply at that period), Vacancies/Takers, and that ratio squared.

Most of the variables in only the retention equation measure family and economic conditions in the civilian labor and housing markets. We expect married sailors to be more likely to remain in the Navy than those who are single. We include the interaction of married and current sea duty. Because a sailor on sea duty receives bonus pay and may or may not prefer being at sea, the sign of this interaction is ambiguous.

How long a sailor has been in the Navy affects reenlistments because a sailor receives a full pension after 20 years of service. A sailor’s length of service is 0 to 6 years and 1 month of service in Zone A; 6 years, 2 months to 10 years, 1 month of service in Zone B; 10 years, 2 months to 15 years in Zone C; 16 to 20 years in Zone D; and more than 20 years in Zone E (less than 1% of sailors). We interact the zone dummies with a sailor’s length of service (months in the military). Thus, these dummies and the interactions allow the length of service to vary in the different zones.

A sailor who is close to having served 20 years, Zone D, is unlikely to leave. To prevent sailors with relatively little time in the Navy from leaving, the Navy sometimes provides a retention bonus to sailors in Zone A in a job that the Navy has difficulty filling. The Navy may provide a smaller bonus for someone in Zone B, but it rarely provides a bonus for sailors in Zone C and does not provide a bonus for more senior sailors.

Time in rank affects whether a sailor stays in the Navy. A sailor who does not receive a quick promotion may choose to leave before hitting the maximum time when the sailor must leave if not promoted. Because the Pass dummy captures whether a sailor has enough time in rank to be eligible for a promotion, we do not include time in rank in the promotion equation.

Base pay is a sailor’s earnings other than bonuses, such as for sea duty or re-enlisting. Pay grade and seniority determine the base pay. The only way a sailor can receive a higher base pay is through promotion. Presumably, sailors compare this base pay to what they think they could earn in the civilian market. However, this variable (expected civilian wage) is highly collinear with one’s pay grade and other explanatory variables in the equation.

To capture general conditions in the civilian labor and housing markets, we added two macro variables lagged one period: The Gross Domestic Product [34] and the unemployment rate [35]. (Initially, we included many more macroeconomic variables, such as the NASDAQ closing index and the mortgage rate, but we found that they had virtually no additional explanatory power).

We also include an estimate of each sailor’s probability of being employed in the civilian labor market, and the expected wage, based on data from the Bureau of Labor Statistics’ Current Population Survey (CPS), the American Community Survey, and the National Longitudinal Survey of Youth (1979 and 1997 cohorts). We estimated an employment-unemployment ML probit equation for the civilian labor market that includes individual characteristics corresponding to those in the Navy data. In the probit, we weighted the data to reflect the over-sampling of veterans in the CPS data. (See the S1 Appendix for details).

The final variable in the retention equation is the (endogenous) promotion dummy. Sailors decide whether to remain in the Navy after learning whether they receive a promotion. During our period, relatively few sailors who receive a promotion subsequently leave: virtually none at E3 and only 5% at E6. The S1 Appendix provide a detailed data dictionary and a further discussion on the data and model used.

## Results and discussion

The Navy had 187 skill groups, which we aggregated into 22 groups. The groups are Administration, Aviation Air Crew, Aviation ATC, Aviation Boatswain, Aviation Mechanical, Aviation Meteorologist, Crypto Intelligence, Diver Special Warfare, Medical, Nuclear, Seabee, Submariner Electronics, Submariner Other, Supply, Surface Combat Electronics, Surface Combat Weapons, Surface Deck, Surface Electrical, Surface Engineering, Surface Operations, and Surface Repair. The various groups involve different tasks, skills, and experience. Therefore, we estimated our model separately for each skill group. (In S1 Fig, we present some basic frequencies from three of these groups.) Because our main results are qualitatively similar across groups and space is limited, we present only the Administration group results. (The S1 Appendix show the main results for the other skill groups).

Administration, a large group, has a bigger share of minorities than in the population and includes sailors with a wide range of backgrounds and AFQT scores. The job descriptions of sailors in Administration include yeoman (administrative and clerical work), personnel specialist, Navy counselor (career and recruiting), musician, mass communication specialist, and legalman (similar to a paralegal).

We start by presenting some basic summary statistics. Then we turn to the main results, including the effects of individual variables on promotion and retention. Next, we discuss decomposing our demographic effects into those due to the promotion process and those due to various groups’ characteristics. The following section examines the effects of specific variables on promotion. Next, we describe the advantage of our model over the usual approach.

### Basic summary statistics

We briefly describe some of the summary statistics, emphasizing demographic group differences.

Averaged over the entire sample period, from 1997 to 2008, the Navy annually promoted 31% of all sailors; 31% of male and female sailors in any given year; 34% of Whites; and 29% of other racial groups. During our sample, nearly all (93%) sailors remained in the Navy, with slight variation across demographic groups. The shares of sailors who were promoted and stayed, promoted and left, and not promoted and left, varied little across demographic groups. However, the shares of sailors who were not promoted and stayed varied across demographics (see S1 Appendix).

Our retention variable indicates whether a sailor remained in the Navy in the period following a promotion. For promotions near the end of an enlistment period, the sailor had to re-enlist to stay in the Navy. However, if the promotion came earlier in the current enlistment period, the sailor remained in the Navy without having to re-enlist. Thus, our retention rates are higher than those that the Navy reports for reenlistments [36].

The racial-ethnic composition varies little across pay grades or time in the service. However, females have substantially less experience than males. For example, 45% of females are in Zone A (0 to 6 years and 1 month of service), but only 33% of males.

The mean AFQT intelligence or ability score is in the 55^th^ percentile for Administration. Whites have a higher mean score, 53%, and Blacks have a lower mean score, 47%, than the other demographic groups. Males have a higher mean score, 56%, than females, 52%.

Blacks and females are more likely to have a high school diploma than other sailors. In Administration, 14% of those in the other races group have some post-high school education, which is at least double the average for the other demographic groups.

Substantially more male sailors, 64%, are married than females, 47%. The share of married Blacks, 56%, is 4 to 5 percentage points lower than for the other racial groups.

More importantly, 39% of sailors have a Pass variable equal to one (not only did they pass the exam, but their Final Multiple exceeds the cut score so that they are eligible for promotion). The share that pass, ranges from 36% for Blacks, 38% for Hispanics, 41% for Whites, and 43% for Others. Both males and females average 39%. Between 55% and 60% of various demographic groups pass the exams the first time.

The fraction of sailors on current sea duty varies relatively little across these groups, with females being 5% more likely to be on sea duty than males. This difference is due to females being more likely to be starting their Navy careers. Over their entire careers, males have spent 47% of their time at sea compared to 40% for females.

Our estimates of the probability that a sailor finds a civilian job range from 92% to 96% for these demographic groups. Blacks, 92%, and females, 93%, have lower probabilities than other groups.

Within a demographic group, the means for most variables differ little between those for all sailors and promoted sailors. One exception is that time in rank is higher for those who receive a promotion. The other exception is time at sea. In any group, promoted sailors average four or five fewer months at sea than the average.

**Fig 2:**
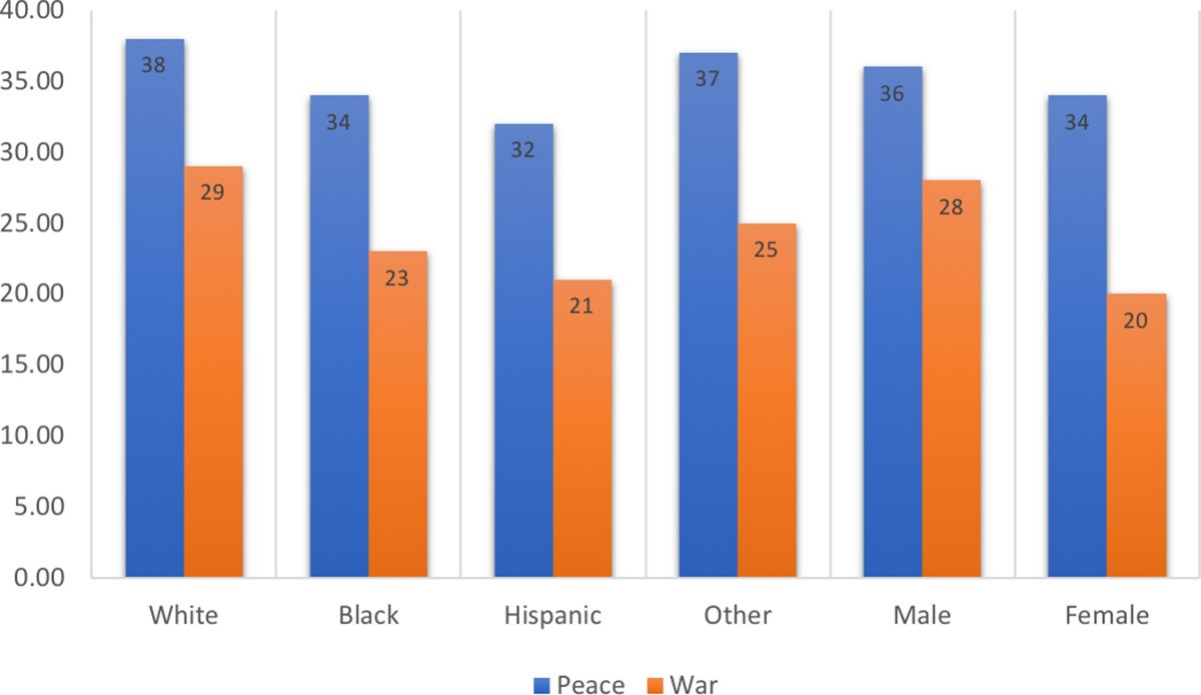
Promotion rates (%) by racial-ethnic groups and sex during peace and war.

Sailors may consider recent promotions and whether they are on the fast track as measured by time in rank when deciding whether to stay in the Navy. The share promoted falls from 79% at E3 and 38% at E4 to about 23% at E5 and E6. The fraction of sailors who remain in the Navy even though they were not recently promoted rises with the pay grade—20% at E3, 59% at E4, 72% at E5, and 76% at E6—because promotions are known to be less frequent at higher ranks (see S1 Appendix and Fig 2).

### Main policy results

Table 1 presents our estimation results. Columns 2 and 3 show the estimates of our bivariate probit model, in which we separately estimate promotion and retention decisions. For comparison purposes, the last column (4) shows the simpler probit model used in many previous studies. As in these earlier studies, the probit’s dependent variable is the joint outcome that an individual receives a promotion and stays in the Navy.

**Table 1:** Bivariate and probit estimation results for administration.

	Bivariate Probit		Probit
Variable	Promote	Retain	Promote and Retain
Constant	1.0242 (0.0735)	2.9872 (2.0461)	7.2940 (0.9640)
AFQT	.0022 (0.0006)	–.0043 (0.0009)	.0051 (0.0007)
High School Diploma	.0124 (0.0292)	.1799 (0.0630)	.1792 (0.0391)
Post High School	.1570 (0.0373)	.1364 (0.0957)	.2572 (0.0548)
Sea Duty	–.0095 (0.0132)	-.0522 (0.0413)	–.2008 (0.0242)
E4 (Pay Grade)	–.8628 (0.0413)	–.0932 (0.1363)	–1.4207 (0.0471)
E5	–1.5512 (0.0409)	.9526 (0.1473)	–3.0227 (0.0555)
E6	–1.9727 (0.0416)	2.5525 (0.1590)	–3.7159 (0.0669)
Black	–.0660 (0.0829)	–.5381 (0.1684)	–.1007 (0.0990)
Hispanic	.1632 (0.1243)	–.3606 (0.2128)	.2304 (0.1383)
Other	.0411 (0.1321)	–.5541 (0.2077)	.1497 (0.1477)
Female	–.1201 (0.0777)	.2005 (0.1407)	–.0383 (0.0883)
Black × E4	–.1510 (0.0561)	.5831 (0.1672)	–.1735 (0.0602)
Hispanic × E4	–.1927 (0.0845)	.3252 (0.2207)	–.2075 (0.0894)
Other × E4	.0046 (0.0927)	.4679 (0.2202)	–.0112 (0.1004)
Female × E4	–.2448 (0.0526)	–.1724 (0.1455)	–.1551 (0.0563)
Black × E5	–.0486 (0.0541)	.7216 (0.1699)	.0915 (0.0611)
Hispanic × E5	–.2564 (0.0839)	.3825 (0.2242)	–.0992 (0.0915)
Other × E5	–.0464 (0.0909)	.7212 (0.2245)	.0914 (0.1003)
Female × E5	–.2281 (0.0524)	–.3139 (0.1472)	–.2396 (0.0586)
Black × E6	–.0960 (0.0554)	.8399 (0.1739)	.3244 (0.0646)
Hispanic × E6	–.2498 (0.0880)	.6189 (0.2337)	.0986 (0.0985)
Other × E6	–.1635 (0.0908)	.9129 (0.2326)	.0289 (0.1019)
Female × E6	.0456 (0.0538)	–.2875 (0.1511)	–.2117 (0.0626)
September 11, 2001	–.0357 (0.0510)	.0458 (0.4181)	1.3173 (0.1713)
Peacetime trend (before 9/11)	–.1922 (0.0388)	.2386 (0.1795)	–.0525 (0.0562)
Peace trend squared	.0301 (0.0066)	–.0422 (0.0294)	.0546 (0.0091)
Wartime trend (after 9/11)	–.4823 (0.0341)	–.6293 (0.0614)	–.4042 (0.0404)
War trend squared	.0313 (0.0052)	.0515 (0.0063)	.0571 (0.0062)
Pass	.7354 (0.0144)		1.0929 (0.0182)
Vacancies/Takers	.3145 (0.0324)		1.1080 (0.1467)
Vacancies/Takers Squared	–.0129 (0.0016)		–1.1977 (0.1757)
Share Sea Duty (fraction)	.6779 (0.1034)	0.5360 (0.0687)	.3583 (0.1332)
Share Sea Duty Squared	–1.5162 (0.1123)		–.2902 (0.1421)
Black × AFQT	–.0000 (0.0009)		–.0029 (0.0011)
Hispanic × AFQT	–.0018 (0.0014)		–.0025 (0.0015)
Other × AFQT	–.0003 (0.0013)		–.0019 (0.0015)
Female × AFQT	.0027 (0.0008)		–.0024 (0.0010)
Black × Peace trend	.0919 (0.0441)		.0975 (0.0496)
Black × Peace trend squared	–.0182 (0.0080)		–.0206 (0.0090)
Black × War trend	.1168 (0.0430)		.1188 (0.0489)
Black × War trend squared	–.0184 (0.0067)		–.0188 (0.0076)
Hispanic × Peace trend	.0228 (0.0664)		.0496 ((0.0736)
Hispanic × Peace trend squared	.0001 (0.0121)		–.0112 (0.0134)
Hispanic × War trend	.0471 (0.0638)		–.0086 (0.0714)
Hispanic × War trend squared	–.0036 (0.0097)		–.0036 (0.0108)
Other × Peace trend	.0168 (0.0714)		.0197 (0.0795)
Other × Peace trend squared	–.0055 (0.0132)		–.0080 (0.0146)
Other × War trend	.0176 (0.0663)		.0299 (0.7573)
Other × War trend squared	–.0026 (0.0098)		–.0097 (0.0114)
Female × Peace trend	.0624 (0.0429)		.0282 (0.0483)
Female × Peace trend squared	–.0123 (0.0078)		.0008 (0.0088)
Female × War trend	–.0643 (0.0408)		.0256 (0.0461)
Female × War trend squared	.0199 (0.0062)		.0033 (0.0071)
Married		.2559 (0.0505)	.1421 (0.0272)
Married × Sea Duty		–.1675 (0.0542)	.0377 (0.0303)
Zone A		–5.2517 (0.2343)	.3317 (0.1395)
Zone B		–5.1929 (0.3016)	.9025 (0.1682)
Zone C		–3.8933 (0.3917)	2.6572 (0.1786)
Zone D		6.3486 (0.4724)	6.0775 (0.2771)
Length of Service × Zone A		.0363 (0.0021)	.0162 (0.0012)
Length of Service × Zone B		.0364 (0.0024)	.0082 (0.0012)
Length of Service × Zone C		.0251 (0.0023)	–.0059 (0.0009)
Length of Service × Zone D		–.0289 (0.0021)	–.0249 (0.0012)
Time in Rank		.0109 (0.0011)	.0254 (0.0005)
Base pay		–.4902 (0.0073)	
GDP		1.1552 (0.1972)	–.7368 (0.0872)
Unemployment. Rate (%)		.6669 (0.0809)	–.0184 (0.0387)
Probability of civilian employment (fraction)		–1.8010 (0.9176)	–2.4864 (0.5399)
Promoted		.9120 (0.0989)	
Ρ	–.3575		
Log-likelihood statistic	–31,148		–18,598
McFadden pseudo R^2^			0.39

Standard errors are given in parenthesis.

Table 2 presents the prediction tables for both models. These models fit the data relatively well.

**Table 2:** The bivariate probit (left panel) and probit predictions table (right panel).

Retained	Promoted			Actual Value	Predicted Value		
	0	1	*Total*		0	1	Total
**0**	1,084	2,700	3,784	**0**	36,791	2,811	39,602
	(2,126)	(353)	(2,479)		(68.7%)	(5.2%)	(73.9%)
**1**	35,818	13,954	49,772	**1**	5,578	8,376	13,954
	(41,149)	(9,928)	(51,077)		(10.4%)	(15.6%)	(26.1%)
**Total**	36,902	16,654	53,556	**Total**	42,369	11,187	53,556
	(43,275)	(10,281)	(53,556)		(79.1%)	(20.9%)	(100%)

Actual vs. Fitted values (Fitted in parentheses) (1 = Promoted and Retained).

Virtually all of the estimated coefficients in Table 1 have the expected signs. We now turn to the four most important results concerning demographic variations in promotion and retention rates.

To keep our presentation short and manageable, we report the results for promotion from E5 to E6. The results are similar for the other steps, though the overall promotion rate decreases with each step. See the S1 Appendix for more results.

Fig 2 shows the conditional promotion rates (in percentages) by demographic groups during peacetime, before 9/11, and wartime, after 9/11. These estimated rates hold the effects of other variables in the equations constant.

*Result 1*: *Nonwhites have lower promotion rates than Whites*, *and females have lower rates than men*. In Fig 2, the blue bars show the estimated promotion rates during peacetime. The promotion rates were 38% for White and 37% for Others (not White, Black, or Hispanic). However, it was only 34% for Blacks and 32% for Hispanics. The promotion rate for males, 36%, exceeds that of females, 34%. Given our large sample, all the differences for the main results we report here and below are statistically significant (we can reject the null hypothesis of no effect at the 0.05 level).

In addition to its short-term impact, a lower promotion rate also affects sailors’ pensions, which depend on their last paygrade. Thus, a lower promotion rate affects sailors for the rest of their lives.

*Result 2*: *Promotion rates for Nonwhites and females were relatively lower during wartime*. The promotion rate differentials for minorities and females became more negative during wartime (red bars). During peacetime, the promotion rate was 4 percentage points lower for Blacks, 6 percentage points lower for Hispanics, and 1 percentage points for Others compared to Whites. The female promotion rate was 2 percentage points below that of males during peacetime. However, during wartime, these differentials doubled, tripled, or quadrupled to 6 percentage points for Blacks, 8 percentage points for Hispanics, 4 percentage points for Others, and 8 percentage points for females.

These promotion differences may seem small, so it is helpful to look at it as the ratios of promotion rate relative to White. For example, in the peace period (before 9/11) the promotion ratio Black/White is about 89%, while during the war period it goes down to 79%. Similarly, for Hispanics these ratios are 84% and 72% respectively for peace and war periods. For Females the ratios (Female/Male) are 91% and 75% respectively for peace and war periods. These are big differences in the promotion rates.

**Fig 3:**
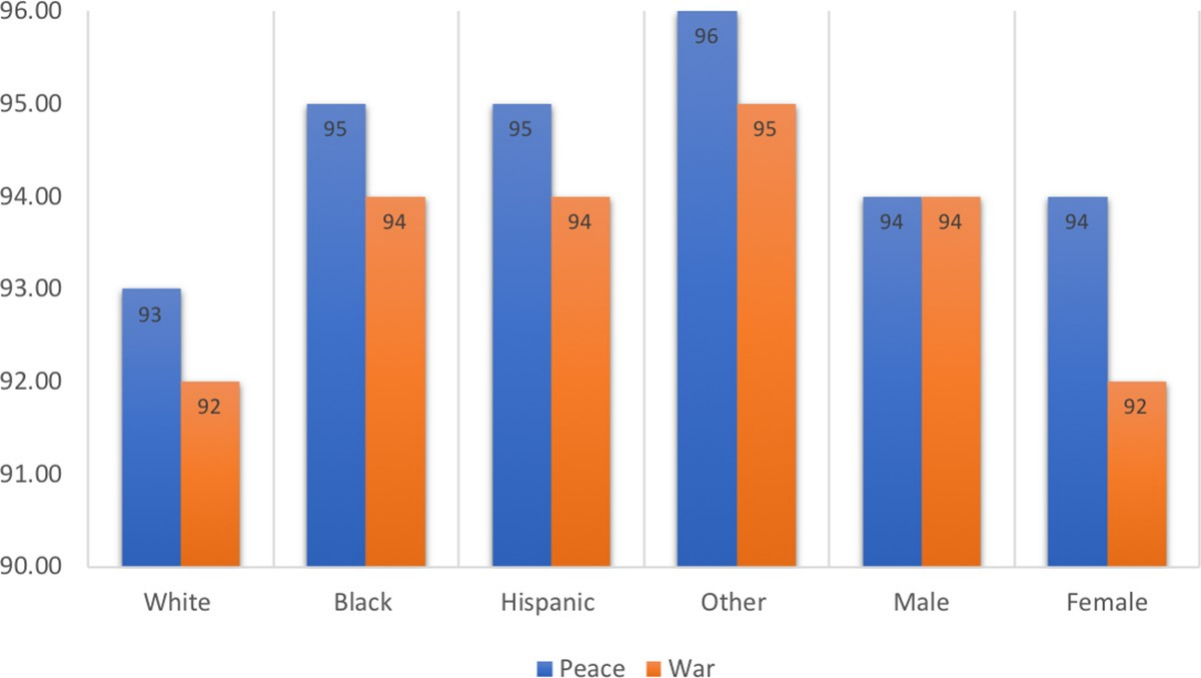
Retention rates (%) by racial-ethnic groups and sex during peace and war.

Fig 3 presents the retention rates by periods and demographic groups.

*Result 3*: *Nonwhites had higher retention rates than Whites during peacetime and wartime*. The retention rates during this period are high and vary by demographic group and over time, as Fig 3 shows. These rates control for other variables, including promotion. Whites have a lower retention rate than do the other racial-ethnic groups. Compared to the White rate, the retention rate was 2% higher for Blacks and Hispanics and 3% higher for Others during peacetime. Overall, the retention rates were lower during wartime, but the race-ethnicity differentials remained the same. Nonwhites may have higher retention rates than Whites because they have fewer options and face more barriers in the civilian labor markets.

*Result 4*: *Females had the same retention rates as men during peacetime*, *but lower rates during wartime*. During peace, the male and female retention rates were both 94%. The male rate stayed the same during wartime, but the female rate fell to 92%. (A 2020 Government Accountability Office study of females in all branches of the military found that the promotion rates were slightly lower for enlisted females and the likelihood of separation was 28% higher than for males [37]).

### Decomposing the effects

Because our bivariate probit is highly nonlinear and has many interaction variables, we cannot infer the role (marginal effects) of specific variables on the probabilities of promotion and retention from the coefficients alone. To show the direct effects of specific variables—particularly race and sex variables—we use two methods of simulation. First, we calculate the expected probabilities over the relevant samples, such as a particular racial group, and then average these probabilities.

Second, we simulate the effect of changing variables on a representative sailor, where we either change a single variable at a time, such as race or gender, or a group of related variables such as Navy policies or civilian labor market conditions. We use our estimated model to calculate each sailor’s expected probabilities and then average these probabilities for each of the two periods. Because the probability of promotion—and to a lesser degree retention—differs substantially before and after 9/11, we report separate simulated probabilities for the two periods. (We provide results of additional skill groups in the S1 Appendix).

Our estimates reflect the substantial change in the Navy’s promotion policies over time. The promotion probability fell by about one-quarter, going from 44.1% in peacetime to 33.8% during wartime. This drop was also due to the long-term plan to shrink the Navy rather than the war.

To illustrate the role of demographic groups on promotion and retention rates during peacetime and wartime, we focus on E5 sailors. These patterns are similar for the other pay grades. The reported retention rates reflect the averaging of different retention rates for promoted and non-promoted sailors. From the beginning of the sample to 2001, the retention probabilities were relatively constant for both groups of sailors. Subsequently, promoted sailors’ retention probabilities fell substantially, while the retention probability of non-promoted sailors rose substantially.

We use simulations and decompositions to show that the effects of coefficients and characteristics on promotion and retention probabilities vary across demographic groups. We simulate average probabilities in two ways. First, we simulate the “actual” probabilities where we use the coefficients for a given group, such as White, to calculate the probabilities for each member of that group and average across them.

Second, we mix and match the coefficients and characteristics other than race (such as education and AFQT) so that we can distinguish between the contribution of coefficients, which may capture unequal evaluations (treatment) for promotion by superiors, and characteristics to the actual differences in probabilities across demographic groups. For example, we ask how Blacks would do if they had the same coefficients as Whites but Black characteristics or if they had the same characteristics as Whites but Black coefficients. This type of analysis is analogous to the [38] decomposition in a linear model.

Table 3 presents these analyses. We start by examining peacetime in columns 3 and 4. As Fig 1 and S1 Fig showed, Whites are more likely to be promoted and less likely to stay in the Navy. These probability gaps reflect both differences in the coefficients between the races and differences in each group’s non-racial characteristics.

**Table 3:** Predicted probabilities (%) by race and sex for E5 sailors.

		Peace (Pre-9/11)		War (Post-9/11)	
Coefficients	Characteristics	Promotion	Retention	Promotion	Retention
**White**	**White**	**37.6**	**92.7**	**29.4**	**91.8**
White	Black	36.5	95.0	25.1	94.4
White	Hispanic	43.0	95.2	24.7	93.9
White	Other	38.2	96.1	25.3	95.1
Black	White	35.0	92.6	27.1	91.7
**Black**	**Black**	**33.7**	**95.0**	**23.2**	**94.3**
Black	Hispanic	34.3	94.9	18.8	93.8
Black	Other	37.7	96.2	23.4	95.1
Hispanic	White	26.6	92.2	24.1	91.6
Hispanic	Black	33.4	91.1	21.3	94.3
**Hispanic**	**Hispanic**	**31.9**	**94.8**	**20.7**	**93.8**
Hispanic	Other	32.8	95.9	21.2	95.0
Other	White	36.6	92.6	28.9	91.7
Other	Black	33.4	95.0	24.7	94.4
Other	Hispanic	36.3	95.0	24.4	93.9
**Other**	**Other**	**37.3**	**96.1**	**24.9**	**95.1**
**Male**	**Male**	**36.0**	**94.3**	**28.2**	**93.6**
Male	Female	33.5	93.5	24.4	92.4
Female	Male	37.0	94.3	22.9	93.5
**Female**	**Female**	**34.0**	**93.5**	**19.5**	**92.2**
**Overall**		**35.5**	**94.1**	**25.7**	**93.2**

The coefficients capture differentials due to possible discrimination within the Navy. In contrast, differentials associated with the demographic groups’ varying characteristics may reflect the limited opportunities for minorities due to systemic racism in society.

The first row of Table 3 presents the conditional probabilities of promotion and retention (columns 3 and 4 respectively) in the peacetime and wartime (columns 5–6) for White sailors. The bold text indicates these numbers are the “actual” estimated probabilities, based on the White coefficients and White characteristics. The promotion probability is 37.6%, and the retention probability is 92.7% during peacetime. These rates drop to 29.4% and 91.8% in wartime.

The next three rows ask what would happen if we use the White coefficients but the characteristics of the other racial/ethnicity groups. The peacetime promotion probability drops from 37.6% with White characteristics to 36.5% for Blacks but rises to 43.0% for Hispanics and 38.2% for Others. That is, if the Navy treated everyone equally in the sense that they had the same (White) coefficients, then the differences in average characteristics would make Blacks less likely to be promoted on average than Whites. However, Hispanics and other races would be more likely to be promoted than Whites. Using the White coefficients, the average probability of retention would be lower for Whites than for the three other racial groups, given their different demographic characteristics.

If we now perform the same type of analysis where we assume everyone has the average characteristics of the White group but the coefficients for their racial group, we find the probability of promotion is 37.6% for Whites, 35.0% for Blacks, 26.6% for Hispanics, and 36.6% for others. Hispanics have, by far, the largest coefficient differences with Whites.

Using this analysis, we can decompose the between-group probability differences into those due to unequal treatment (coefficient differences) and those due to differing characteristics. For example, for E5 sailors during peacetime, the actual difference in promotion probabilities between Whites and Blacks was 3.9 percentage points (= 37.6%– 33.7%). However, the difference using White coefficients and the corresponding characteristics is only 1.1 percentage points (= 37.6%– 36.5%), while the difference using White characteristics and varying coefficients is 2.6 percentage points (= 37.6%– 35.0%). Thus, *Result 5*: *Differences in coefficients—which may capture unequal evaluations by superiors—is roughly twice as important as differences in characteristics in explaining the overall difference in promotion probabilities between Whites and Blacks*.

The comparable percentage point differentials between Whites and Hispanics are 5.7 percentage points (overall), –5.3 percentage points (varying characteristics), and 11.1 percentage points (varying coefficients). One possible interpretation of these results is that the “superior” average characteristics of Hispanics cuts in half the difference in promotion probabilities due to unequal treatment by Navy supervisors. Thus, *Result 6*: *Hispanics may face the most adverse treatment of any demographic/ethnic group*.

How did the race-ethnicity differentials change during wartime? The actual gap in promotion probabilities between Whites and Blacks went from 3.9 percentage points before 9/11 to 6.1 percentage points after 9/11, from 5.7 percentage points to 8.7 percentage points for Hispanics, and from 4.3 percentage points to 4.5 percentage points for others. These increased promotion gaps are even more striking, given that the probability of promotion fell for all groups.

If we hold characteristics fixed using the average for the White group and vary coefficients, the difference in promotion probabilities was 2.6 percentage points for Blacks, 11.0 percentage points for Hispanics, and 1.0 percentage points for Others before 9/11. The corresponding differentials for the post 9/11 period are 2.3, 5.3, and 0.6.

The increase in the actual differential must be due to changes in the various groups’ relative characteristics. If we use the White coefficients and vary characteristics across racial groups, the difference in promotion probabilities was 1.2 percentage points for Blacks, –5.4 percentage points for Hispanics, and –0.6 for others during peacetime. The corresponding differentials during war are 4.3, 4.7, and 4.1. The primary reason why the actual differentials between Whites and other groups have increased over time is due to relative changes in the characteristics of Nonwhite groups. These changes in characteristics are so large that they swamp the coefficient effects that go in the other direction.

The changes in retention probabilities over time are much smaller than for promotion probabilities. The probability of staying in the Navy fell by 0.9 percentage points for Whites, 0.7 for Blacks, and 1.0 for Hispanics and others.

Gaps in the promotion probabilities between the races are qualitatively similar between pay grades, but the gaps vary. During the war period, Whites are 8.5 percentage points more likely to be promoted than Blacks in E4, 6.2 percentage points more likely at E5, and 8.2 percentage points more likely at E6.

The bottom four rows of Table 3 report a similar analysis for men and women. Again, we focus on E5 sailors. As we did above, we decompose probability differences into those based on coefficient differences and those based on differences in characteristics other than sex.

The actual differential between men and women was 2.0 percentage points during peacetime. Using male coefficients and each sex’s characteristics, we calculate the differential as 2.5 percentage points. Using characteristics of males and varying the coefficients, the differential was –1.0 percentage point. One possible interpretation is that the Navy slightly favored females, but differences in characteristics between males and females caused men to be more likely to be promoted.

The corresponding differentials during the war period are 8.7 percentage points (actual), 3.8 percentage points (varying characteristics), and 5.3 percentage points (varying coefficients). These results imply that the actual E5 gap between men and women increased because the Navy went from favoring women to favoring men, and the average characteristics of women changed adversely relative to those of men. The S1 Appendix provide further details on the differences between the different pay grades.

As the S1 Appendix show, we observe a qualitatively similar pattern for E4, but a strikingly different one for E6. The E6 differentials were –3.2 percentage points (actual), –2.4 (varying characteristics), and –0.7 (varying coefficients) during peacetime; and are –0.8, 1.4, and –2.2 during wartime. Thus, it appears that the Navy favored E6 women during peace and even more so during war. However, women had relatively more important (for the Navy) characteristics during peacetime than during wartime. The net effect was that E6 women were much more likely than men to receive a promotion during peacetime but only slightly more likely during wartime.

For E5, men were 0.9 percentage points more likely to stay in the Navy than women based on the actual probabilities during peace. Using men’s coefficients and varying the characteristics results in the same differential, 0.9. Using male characteristics and varying the coefficients eliminates the differential. Corresponding differentials during war are 1.4 percentage points (actual), 1.2 percentage points (varying characteristics), and 0.1 percentage points (varying coefficients). Thus, the pattern of differentials does not vary much over time.

What do these simulations tell us? The probabilities of promotion and retention could differ across demographic groups for three reasons. First, the Navy treats demographic groups differently in the sense that people with the same characteristics other than race or sex have different promotion and retention probabilities. Second, demographic groups have different mixes of observed characteristics, such as education, experience, and civilian market opportunities. Third, demographic groups possess different unobserved characteristics.

We cannot explicitly examine the third hypothesis about unobserved characteristics. However, because our estimation model includes an objective ability measure, the AFQT score, and many other observed characteristics and controls, it is unlikely that racial and sex differences in promotion rates reflect significant unmeasured ability differences across demographic groups.

### Effects of specific variables on promotion and retention

We conduct simulation experiments for an Administration-group sailor with the overall sample means to learn how specific variables affect the promotion and retention probabilities. This typical sailor is married, a high school graduate, in pay grade E4, currently is on shore duty, has an AFQT of 56, 105.9 months of service, 31.6 months in rank, spent 42.7% of his or her Navy career at sea, is eligible for promotion, has a pass rate on the exam of 37.1%, has a base pay of $26,746, and has an estimated probability of civilian employment of 94.5%. In 1998, the base period, the ratio of Vacancies to Takers was 0.32, the civilian unemployment rate was 4.9%, and the annual GDP was $9.52 trillion.

Table 4 presents these simulated results. The columns show how the promotion probabilities vary across demographic groups. The rows illustrate how the probability of promotion changes relative to the base case as we change one variable at a time (say, White male to White female). The first row shows the probabilities of promotion for the base characteristics by race and sex. The number in the first column of the first row, 60.7%, is the promotion probability for a White male. The second column of the first row shows that a White female’s probability of promotion was only 49.5%, even though she has the same characteristics as her male counterpart. That is, she has a 11.2 percentage points lower promotion probability.

**Table 4:** Promotion simulations (%).

	White Male	White Female	Black Male	Black Female	Hispanic Male	Hispanic Female
**Base Case**	**60.7**	**49.5**	**53.6**	**42.4**	**59.2**	**48.0**
AFQT = 80	62.9	51.7	55.9	44.6	61.5	50.2
AFQT = 35	59.2	47.8	52.0	40.8	57.7	46.3
Post HS	66.1	55.1	59.3	48.0	64.7	53.6
1.5×Vacancies/Takers	61.7	50.5	54.7	43.4	60.3	49.0
Post 9/11	43.9	36.9	37.4	30.9	42.5	35.6
E5	34.1	25.0	31.3	22.6	30.5	21.9

*Notes*: Base case characteristics: married, high school graduate, E4, on shore duty, share of career at sea = 42.7%, AFQT = 56, service = 105.871 months, time in rank = 31.61 months, eligible for promotion, pass rate = 37.1%, base pay = $26,746, estimated probability of civilian employment = 94.5%, year = 1998, vacancies/takers = 0.318, civilian unemployment rate = 4.92%, and GDP = $9.52 trillion.

The third column shows that a Black male, with the same characteristics as a White one, has a promotion probability of only 53.6%—7.1 percentage points less than for a White male. The promotion probability of a black female is 7.1 percentage points less than a White female and 18.3 less than a White male. Thus, the differences are enormous.

Moving down the rows, we see how the promotion probability changes for a given demographic group as we change one variable at a time. The first row of the first column shows the base case: A White male, with the mean AFQT score (56), has a promotion probability of 60.7%. The second row shows that raising the sailor’s AFQT score to 80, holding everything else constant, increases his probability of being promoted by 2.2 percentage points to 62.9%. The other columns show that this increase in the AFQT score raises each demographic group’s probability of promotion by 2.2 to 2.4 percentage points. Similarly, the third row shows that lowering the AFQT to 35 reduces the promotion probabilities for the various demographic groups, but by smaller absolute amounts than raising it.

Having some post-high school education raises the probability of promotion between 5.4 to 6.1 percentage points. As we control for intelligence as measured by the AFQT, presumably, a sailor with post-high school education is rewarded because of some extra knowledge or better discipline associated with the extra education.

The probability of promotion fell substantially over the last half of the sample period. For a White male, the probability was 60.7% during peacetime and 43.9% during wartime. For a Black female, the probability dropped from 42.4% to 30.9%, respectively.

Similarly, an increase in rank lowers the probability of a promotion. A typical White male’s probability is 60.7% at E4, but only 34.1% at E5 (row 7). Again, the promotion probabilities (E5) are lower for Nonwhites and females.

Although the table does not show them, the retention probabilities also vary with characteristics, but to a lesser degree than promotions. One particularly striking, plausible insight is *Result 7*: *The higher a sailor’s AFQT*, *the more likely the sailor receives a promotion*, *but the less likely the sailor stays in the Navy*.

When civilian labor markets have poor conditions, sailors are more likely to remain in the Navy. As shown in the S1 Appendix, the simulated results show that Blacks and Hispanics (males and females) have higher probabilities of staying if not promoted relative to their White counterparts. These results hold for both periods. Presumably, this result reflects that Blacks and Hispanics have fewer civilian opportunities than Whites due to systemic discrimination in the labor market. These results are also consistent with the detailed analyses of the conditional probabilities of promotion and retention presented in Table 3 and in the supplement. For example, the average (across paygrades during the peace period) of staying in the Navy if not promoted is 52.6% for Whites. For Blacks and Hispanics it is 57.2 and 56.1 respectively. Thus, *Result 8*: *Blacks and Hispanics are less likely than Whites to be promoted*, *and more likely to stay in the Navy if not promoted*.

In general, during slack conditions in civilian labor markets, sailors are more likely to remain in the Navy. For example, if the civilian unemployment rate increases from 4.9% to 6.3%, the probability that a White male remains rises by 0.2 percentage points if promoted and by 0.3 percentage points if not promoted. If that sailor’s estimated individual probability of finding civilian employment falls from 94.5% to 75%, a White male’s probability of remaining in the Navy rises by 0.1 percentage point if promoted and by 0.2 percentage point if not promoted. These quantitative effects are small because virtually all E4 sailors remained in the Navy in 1998. The quantitative effects are larger for more recent years, though the qualitative effects are the same.

Thus, our overall conclusion to this last discussion is *Result 9*: *Individual characteristics affect promotion and retention rates*, *but race and sex play the dominant roles*.

### Modeling advantages

Our analysis uses a bivariate probit model, where we simultaneously estimate the sequential decisions of the employer (the Navy) and the employees (sailors). In contrast, most previous promotion studies looked at the combined outcome a worker was promoted and stayed with the firm. To show how estimating this more restrictive model affects results, we compared our results to one that most previous studies typically used. The last column of Table 1 shows our estimates of a the classical binary probit model where the dependent variable is one for an individual who is promoted and retained and zero otherwise.

Combining the promotion and retention effects into the single outcome (promote and stay) masks the offsetting effects in the bivariate model. Thus, any variable with opposite signs in our promotion and retention bivariate probit model may have little or no net effect in the probit model. For example, in our model, having a higher AFQT score raises the probability of being promoted and lowers the probability of staying. Here, the net effect in the probit model is positive.

Another example is an E6 Hispanic sailor who is less likely to be promoted but more likely to stay than a White sailor in the bivariate model. In the probit model, the Hispanic sailor does not have a statistically significant difference to a White counterpart in being promoted and staying. Other race variables are similar. Thus, the probit model does not capture patterns—particularly those involving race—in the same way as our general two-decision bivariate model.

## Conclusions

The Navy has made a serious attempt to prevent discrimination in promotions by using formal rules, policies, and its evaluation process. The complex evaluation process relies primarily, but not exclusively, on objective criteria. Although the Navy can objectively measure sailors’ knowledge, experience, and other factors, it must also evaluate how well sailors perform their jobs. That evaluation by a sailor’s superior is subjective. It is difficult to imagine how such reviews can be entirely objectively quantified. Unfortunately, this one subjective aspect of the process, which has a significant weight in the promotion formula, has led to unequal promotion and retention rates by race/ethnicity and sex. Because we do not have information about the race and sex of sailors’ supervisors, we do not know how that affects evaluations.

We draw nine main conclusions. First, holding everything else constant, Nonwhites have lower promotion rates than Whites, and females have lower rates than men. Second, promotion rates for Nonwhites and females were relatively lower during wartime. Third, Nonwhites had higher retention rates than Whites during peacetime and wartime. Fourth, females had the same retention rates as men during peacetime, but lower rates during wartime.

Fifth, differences in how the Navy promotes sailors affect Black and White differential promotion rates by twice as much as differences in the groups’ characteristics. Sixth, Hispanics face the most discrimination of any demographic group. Seventh, the higher a sailor’s measured ability (AFQT), the more likely the sailor receives a promotion, but the less likely the sailor stays in the Navy. Eighth, Blacks and Hispanics are less likely than Whites to be promoted, and more likely to stay in the Navy if not promoted. Ninth, individual characteristics affect promotion and retention rates, but race/ethnicity and sex play the dominant roles.

These results point toward unequal treatment of sailors by race and sex. Further implications of our results are that the lower promotion rates for Nonwhites and female enlisted sailors affects their wages throughout their service and their retirement years.

Our promotion and retention study is the first to consistently estimate the sequential promotion decision of the Navy and the retention decisions of sailors, accounting for the feedback between them. These differences allow us to detect effects that previous studies that only looked at the joint outcome of promotion and retention missed. Additionally, we are better able to control for other factors than race/ethnicity or gender than in previous studies. The Navy’s superior data allows us to control for a sailor’s ability and individual-specific information. It is also the first study to carefully model employees’ options in other (civilian) labor markets. These extra control variables result in more accurate estimates of the role of race, ethnicity, and sex on promotions and retention.

## Supporting information

S1 Appendix Supplementary materials. (DOCX)

S1 Fig Relative promotion and ‘not promoted but stayed’ (E5 to E6) frequencies of Blacks, Hispanics, and females for three different Navy skill groups. (TIF)
