# Supplementary material for: Does the U.S. Navy’s reliance on objective standards prevent discrimination in promotions and retentions?
Source: PLoS One. 2021 Apr 28;16(4):e0250630. doi: 10.1371/journal.pone.0250630 (PMC8081226; doi:10.1371/journal.pone.0250630)
Supplement: S1 Appendix — (DOCX) [file pone.0250630.s001.docx]

**S1 Appendix. Supplementary materials**

**Detailed data dictionary**

The first set of variables in this supplementary material includes all the dummy variables. Most of these variables were created to reflect special events in the sailor’s history, or to capture some of the basic questions of interest concerning geographic stability, impact of transfers, or impact of job or sea duty rotations. In general, the fleet concentration variables reflect basic geographic location and specific (unobserved) characteristic of specific fleets. Note that Fleet Concentration areas are very large geographic areas. We use the set of variables FLCONC as dummy variables for each one of the possible fleet concentrations. These fleet concentrations are varied by skill group. The ATC variables reflect the exact geographic location of the Sailor’s duty station during the observed time period. The EXAM variables reflect the number of times a sailor took the pre-promotion examination for her/his grade. These variables capture the “speed” of promotion from the time the sailor is eligible for promotion and provide an indirect measure of the individual’s standing within her/his cohort. The variable NAR has a value of one if the sailor is eligible for promotion within her/his pay grade. The set of variables called DNEC (Distributable Navy Enlistment Code - Distributed NEC/skills) provide a proxy for specific requirements for a given job. The Navy has over 2000 NEC’s, and we investigated those that might affect the career paths of individuals). In most cases, we use only two dummy variables to capture education (high school education and above high school education). The reason for that is that in the enlisted skill groups only a very small number of individuals have higher education. In special cases (highly skilled occupations), where such training is more common, we use more detailed measures. Finally, whenever possible we use gender and race dummies as well. The different races are Black, Hispanic, Other and White.

The next set of variables includes continuous variables. The AFQT stands for the Armed Forces Qualifications Test, a test given to new recruits in the U.S. armed forces. The possible range is 30 through 99, where 30 is the lowest score possible. This variable captures the basic ability (overall quality) of an individual. The months at sea (SEAMONTH) variable captures the overall number of months at sea. In the estimation model we normalize this variable to Seamonth/LOS where LOS is the overall length of service for that sailor. The TIR is the time (months) in grade since last promotion. The VACANTS variable is the Navy demand (open vacancies) at that time for that pay grade and a specific skill specialization. (For each exam cycle, VACANTS is the approximate number of vacancies for the rating – pay grade combination to which the Sailor was attempting to be promoted.) The TAKERS reflect the total supply (number of individuals, who are potentially up for promotion) at that period, pay grade and skill. (For each exam cycle, the approximate number of Sailors who had the opportunity to advance.) In our model we use the variable Vacants/Takers. PMA stands for performance mark average and reflects an overall (subjective) evaluation of that individual by her/his supervisor/s. The PNA variable reflects Actual Passed Not Advanced points (points a sailor that is eligible for promotion, passed her/his pre-promotion exam, but was not promoted). The CYCLECUT variable reflects a value corresponding to the TAKERS (demand) value that determines whether an individual will be promoted at that cycle period. If the individual’s score (INDSCORE) is above that value, the individual is high enough within her/his cohort and will be promoted. That is, if INDSCORE is greater than CYCLECUT the individual will be promoted (at that period). The MULTIPLE variable is a variable we create in order to imitate the Navy’s promotion decision process. Following the Navy’s guideline the pay grade-specific multiple for each individual at each promotion period is calculated. This MULTIPLE is a weighted average of other performance variables, the time in current grade and a variable (PNA) reflecting points received for successfully completing the pre-promotion examination, but then (due to not enough vacancies) not being promoted. This multiplier value is changed at each promotion cycle. Finally, the set of macroeconomic indicators is self-explanatory.

These supplementary materials provide a list and definitions of the complete set of variables used in this paper. For each of the skill groups there are a set of fixed covariates as well as a set of skill group specific covariates included in the models. The following table describes these variables. We provide also the “source” meaning how/where these variables are created. We use the word “raw” to indicate it is in its original state received in the original data sets, and “created” means we created it via variables’ manipulations prior to the estimation phase.

**Table S1. Basic variables’ definitions.**

| **Variables** | **Definitions (Description/Source/Instructions/Notes and Computer codes)** |
| --- | --- |
| ***Dummy variables*** | |
| CS (3-8) | The sailor’s current state (pay grade E3-E7, or Loss with Loss=8) |
| LS (3-6) | The sailor’s lagged state (pay grade at the end of the previous year) |
| HS | *Label*: Whether or not the sailor has completed high school.  *Source*: Raw |
| MTHS | *Label*: Whether or not the sailor has education higher than high school.  *Source*: Raw |
| MARRIED | *Label*: Whether or not the sailor is currently married.  *Source*: Raw |
| FLAG_TR | *Label*: Whether or not the sailor has ever been transferred.  *Source*: Created  *Instructions*: This is a flag that is set to 1 if the sailor has ever been transferred and 0 if never been transferred. |
| FCC_PROM | *Label*: Whether or not the sailor has changed Fleet Concentration since last promotion (or since start of current pay grade).  *Source*: Created  *Instructions*: Scan the Sailor’s records since the last promotion (or since the beginning of the current pay grade) and set this flag to 1 if there is any change in Fleet Concentration found. If not, set this flag to 0. |
| SS_SEA | *Label*: Is the Sailor currently at sea.  *Source*: Raw |
| SS_OTH | *Label*: Is the Sailor’s current Seashore code is something other than SEA or LAND.  *Source*: Raw |
| SSC_LO | *Label*: Whether the sailor never changed from Sea to Shore duty or if (s)he changed once, since the sailor was first observed in the data.  *Source*: Created  *Instructions*: Scan the sailor’s complete history for changes in Sea Shore duty codes and set this flag to 1 if the sum of these changes is 0 or 1. Else, set the flag to 0. |
| SATCC_LO | *Label*: Whether the sailor’s ATC code did not change since the last promotion or start of current pay grade.  *Source*: Created  *Instructions*: Scan the Sailor’s record for change in ATC (since the last promotion or start f this pay grade) and set this flag to 1 if no ATC changes are found. |
| SATCC_ME | *Label*: Whether the sailor’s ATC code has changed just once since the last promotion or start of current pay grade.  *Source*: Created  *Instructions*: Scan the Sailor’s record for change in ATC (since the last promotion or start f this pay grade) and set this flag to 1 if only 1 ATC change is found. |
| EXAM_M | *Label:* If the sailor has been eligible for this promotion 2 or 3 times (the sailor took the exams more than once in the past).  *Source:* Created  *Instructions:* Scan the Sailor’s record for the relevant event_id to see how many times has (s)he been eligible for promotion. The scans are pay grade specific. For example, if the sailor is currently at E3, and is eligible for E4, then scan the sailor’s record to see how many times event_id 9 occurred in his/her record (since the last promotion). Set this flag = 1 if the current count is equal to 2 or 3 else set this flag to 0. The scanning is done through the last month of the current period. |
| EXAM_S | *Label:* If the sailor has been eligible for this promotion 4 or more times.  *Source:* Created  *Instructions:* Scan the Sailor’s record for the relevant event_id to see how many times has (s)he been eligible for promotion. The scans are pay grade specific. For example, if the sailor is currently at E3, and is eligible for E4, then scan the sailor’s record to see how many times event_id 9 occurred in his/her record (since the last promotion). Set this flag = 1 if the current count is equal to 4 or more else set it to zero. The scanning is done through the last month of the current period. |
| NAR | *Label*: Whether or not the sailor is eligible for promotion (i.e., passed her/his minimum time in pay-grade).  *Source*: Created  *Instructions*: If a non-missing VACANTS variable is recorded to the current observation, then set this flag to 1, else set it to 0. |
| FLCONC00  FLCONC01  …  FLCONC11 | *Label*: Sailor’s current Fleet Concentration.  *Source*: Raw  *Note*: In the actual models, when a particular FLCONC category is very rare for a given skill group (i.e., less than 2%) it is not included in the models.  Fleet Concentration Area --  0 NON FLEET CONCENTRATION AREA  1 NORFOLK  2 JACKSONVILLE  3 NEW LONDON  4 SAN DIEGO  5 HAWAII  6 PACIFIC NORTHWEST  7 LEMOORE  8 BRUNSWICK  9 CORPUS CHRISTI  10 GULFPORT  11 PORT HUENEME |
| DNECXXXX | *Label*: Sailor’s DNEC code.  *Source*: Raw  *Note*: In the actual models, when a particular DNEC category is very rare for a given skill group (i.e., less than 2%) it is not included in the models. The DNEC categories are skill group specific. |
| ***Continuous Sailor and Promotion Cycle Specific Variables*** | |
| AFQT_N | *Label*: Sailor’s AFQT Score.  *Source*: Raw  *Note*: All sailors with AFQT Score less than 30 or higher than 99 are removed from the data. |
| SEAMONTH | *Label*: Number of months, to date, that the Sailor has spent continuously at Sea.  *Source*: Raw |
| SEAMONTH2 | *Label*: SEAMONTH Squared  *Source*: Created  *Instruction*: This is just the square of the raw variable SEAMONTH |
| LOS | *Label*: Sailor’s Length of Service, to date.  *Source*: Raw |
| LOS2 | *Label*: Sailor’s Length of Service Squared  *Source*: Created  *Instructions*: This is just the square of the raw variable (LOS) |
| TIR | *Label*: Sailor’s Time in Current Rank, to date.  *Source*: Raw |
| TIR2 | *Label*: Sailor’s time in current rank squared  *Source*: Created  *Instructions*: This is just the square of the raw variable (TIR) |
| VACANTS | *Label*: Approximate number of vacancies for the rating-pay grade combination to which the sailor is attempting to be promoted (exam cycle specific).  *Source*: Raw |
| TAKERS | *Label*: Approximate number of sailors who had the opportunity to advance (exam cycle specific).  *Source*: Raw |
| PMA | *Label*: Most recent Performance Mark Average value.  *Source*: Raw |
| CYCLECUT | *Label*: Minimum score used by promotion period to determine advancement.  *Source*: Raw |
| INDSCORE | *Label*: Sailor’s individual final multiple score for the latest promotion cycle.  *Source*: Raw |
| MULTIPLE | *Label*: Sailor’s pay grade specific Multiple Score  *Source*: Created  *Instructions*: This variable is created using the following pay grade specific calculations.  **For E3 and E4**: MULTIPLE = 0.34*INDSCORE + 0.36*((PMA*60) - 156) + 0.13*((TIR*2) + 15) + 0.13*(2*PNA)  **For E5**: MULTIPLE = 0.30*INDSCORE + 0.415*((PMA*60) - 130) + 0.13*((TIR*2) + 19) + 0.11*(2*PNA)  **For E6**: MULTIPLE = 0.60*INDSCORE + 0.40*(PMA*13) |
| ***Continuous Macroeconomic Variables*** | |
| MTGAGE_1 | *Label*: Mortgage rate from one month prior to observation.  *Source*: Raw |
| UNEMPL_1 | *Label*: Unemployment Rate from one month prior to observation.  *Source*: Raw |
| R_GDP_1 | *Label*: Real GDP from one month prior to observation.  *Source*: Raw |
| NASDAQ_0 | *Label*: NASDAQ closing index for the month of this observation.  *Source*: Raw |

Data Notes:

1. The maximum is 10 years for E4, 20 years (reduced to 14 in 2005) for E5, 20 years for E6, and 24 years for E7.

We do not include the Performance Mark Average (PMA), Pass not Advance (PNA), and the Final Multiple score because they may be a function of the race and sex dummies, which are included in the equations. The PMA (and hence the Final Multiple) is a subjective evaluation of an individual by the sailor’s supervisor, who might be partially influenced by the sailor’s race or sex. To test for possible subjectivity, we regressed PMA and the Final Multiple Score on demographic variables. Many of the coefficients on race and gender were statistically significantly different from zero.

The share of sailors who take and pass the promotion exam the first time is 58% for males, 56% for females, 60% for Whites, and 55% or 56% for Blacks, Hispanics, and other races.

**Civilian wage estimation**

The civilian data came from four sources. All of these data sets are used in the estimation phase. The four data sets are:

1. American Community Survey (ACS, updated 2007).
2. The Current Population Survey (CPS, March Supplement, 2008).
3. The National Longitudinal Survey of Youth (1979 and 1997 cohorts). These two data sets are much smaller but contain information that does not exist in the other data sets such Aptitude tests, AFQT values and background information on each individual. Further, these data allow us to study the behavior of individuals (veterans in particular) over time. These data sets are used to study veterans’ behavior, major occupations taken by veterans and allowed us to capture the effect of AFQT on wages and employment probabilities.
4. Macroeconomic indicators (in current and lag values). Annual, quarterly and monthly data are used.

The table below provides the complete list (and definitions) of variables used for the wage estimation. These variables are used to estimate (separately) each civilian occupation. If a certain variable cannot be used or is statistically insignificant, it is not used.

**Modeling Note:**

We cannot use a Heckman-type employment probit-wage equation model because the resulting estimated wage for a sailor would be a linear function of the sailor’s personal characteristics, which are already included in the retention equation, and hence nearly perfectly collinear. The unemployment estimate is a highly nonlinear function of these characteristics; hence it does not pose the same collinearity problem. See supplemental material.

**Table S2. Basic variables’ definitions.**

| **Variable definitions** | |
| --- | --- |
| EMPLOYED | 1 = employed, 0 = unemployed |
| RWAGE | Wage and salary income in 2006 dollars |
| EMPLOYED | 1 = Employed |
| LOGWAGE | Log of Real WAGE |
| EXPER/LEXP | Experience as defined by our formula/exp minus 1; Formula, IF (AGEP > 15) THEN EXPER = AGEP - (10*NODIP) - (12*HSDIP) - (13*SOMECOL) - (14*AADEG) - (16*BADEG) - (19*PROFDEG) - (21*GRADDEG) - 6; |
| EXPERSQ/LEXPSQ | Experience Squared/ exp minus 1 squared |
| HISP | 1 = Hispanic, 0 = other |
| VETSTAT | 1= Veteran |
| GENDER | 1 = male, 0 = other |
| MARITAL | 1 = married, 0 = not married |
| VETSTAT | 1 = veteran 0 = non-veteran |
| VETEXP | Veteran’s experience, in years |
| NOHS | 1 = No HS diploma |
| HSDIP– reference group | 1= HS diploma |
| SOMECOL | 1 = Some college |
| AADEG | 1= Associate degree |
| BADEG | 1 = BA degree |
| PROFDEG | 1= Prof degree |
| GRAGDEG | 1 = Graduate degree |
| PGDEG | 1 = Prof & Graduate degree |
| WKHP | Worked hours per week, in hours |
| WKW | Worked weeks per year, in weeks |
| HWY | Hours Worked, Yearly = WKHP x WKW |
| AGE1-AGE5 – AGE1 is reference group | Age groups 16-23, 24-33, 34-43, 44-59, and 60-65 |
| A1NOHS – A5NOHS | Education (NOHS) interacted with AGE1-AGE5 |
| A1HS-A5HS | Education (HS) iinteracted with AGE1-AGE5 |
| A1ASSOC – A5ASSOC | Education (ASSOC) interacted with AGE1-AGE5 |
| A1SOMCOL – A5SOMCOL | Education (SOMECOL) interacted with AGE1-AGE5 |
| A1BA – A5BA | Education (BA) interacted with AGE1-AGE5 |
| A1PROF – A5PROF | Education (PROF) interacted with AGE1-AGE5 |
| A1GRAD – A5GRAD | Education (GRAD) interacted with AGE1-AGE5 |
| A1PGDEG – A5PGDEG | Education (PROF &GRAD) interacted with AGE1-AGE5 |

**Wage equation estimation**

We estimated the civilian wage (not by occupation) using the ACS data and weights based on the CPS data. The data used are limited to the working age population (ages 16 through 65) and for individuals in the work force, whether employed or unemployed. The data exclude individuals with reported self-employment income. These data were weighted to resemble the CPS March Supplement data which is a correct sample of the underlying U.S. population and reflects the overall unemployment rate.

Since our interest is in the veterans, rather than using a “perfect” sample, we used a sample with oversampling the number of veterans. The final sample used included all employed and unemployed veterans (*n* = 210,171), a random sample of employed civilians (n = 180,000), and a random sample of unemployed civilians (*n* = 30,000). The final sample consists of 420,171 individuals. In this sample 50 % are veterans and 9.74 % are unemployed. Tables S3A and S3B provide the estimated results (both the first part, the selection equation, and the second part, the wage equation) of the weighted Heckman two-stage estimation.

**Table S3A. Probit selection model with all occupations; *n* = 420,171.**

| **Parameter** | **Estimate** | **Standard Error** | **Pr > ChiSq** |
| --- | --- | --- | --- |
| **Intercept** | 0.9607 | 0.0111 | <.0001 |
| **Experience** | 0.0053 | 0.0020 | 0.0099 |
| **Experience Squared** | 0.0000 | 0.0000 | 0.3448 |
| **Black** | -0.3672 | 0.0094 | <.0001 |
| **Hispanic** | -0.0854 | 0.0085 | <.0001 |
| **Gender (M=1)** | 0.0948 | 0.0073 | <.0001 |
| **Marital** | 0.2467 | 0.0076 | <.0001 |
| **No HS** | -0.3054 | 0.0095 | <.0001 |
| **Some College** | 0.3592 | 0.0175 | <.0001 |
| **Assoc. Degree** | 0.5682 | 0.0462 | <.0001 |
| **BA Degree** | 0.4646 | 0.0193 | <.0001 |
| **Professional Degree** | 0.4986 | 0.0429 | <.0001 |
| **Graduate Degree** | 0.4340 | 0.0195 | <.0001 |
| **Veteran** | 0.3633 | 0.0269 | <.0001 |
| **Veteran Exp. (Years)** | 0.0016 | 0.0027 | 0.5523 |
| **Veteran Exp. Squared** | -0.0002 | 0.0001 | 0.0142 |
| **Age Group 2** | 0.2648 | 0.0173 | <.0001 |
| **Age Group 3** | 0.3152 | 0.0261 | <.0001 |
| **Age Group 4** | 0.3178 | 0.0332 | <.0001 |
| **Age Group 5** | 0.3036 | 0.0502 | <.0001 |
| **Age 2 – Associate (interaction)** | -0.2224 | 0.0533 | <.0001 |
| **Age 2 - SomCol (interaction)** | -0.2048 | 0.0248 | <.0001 |
| **Age 3 – Associate (interaction)** | -0.3284 | 0.0524 | <.0001 |
| **Age 3 - SomCol (interaction)** | -0.1968 | 0.0261 | <.0001 |
| **Age 3 – BA (interaction)** | -0.0885 | 0.0274 | 0.0012 |
| **Age 4 – Associate (interaction)** | -0.3549 | 0.0516 | <.0001 |
| **Age 4 – SomCol (interaction)** | -0.2544 | 0.0246 | <.0001 |
| **Age 4 – BA (interaction)** | -0.2057 | 0.0266 | <.0001 |
| **Age 5 – No HS (interaction)** | 0.1690 | 0.0561 | 0.0026 |
| **Age 5 – Associate (interaction)** | -0.4510 | 0.0855 | <.0001 |
| **Age 5 - SomCol (interaction)** | -0.2541 | 0.0561 | <.0001 |
| **Age 5 – BA (interaction)** | -0.3526 | 0.0582 | <.0001 |
| **Log Likelihood** | -82542.4478 |  |  |

Variables’ Definitions are in Table S1.

**Table S3B. Log wage equation with all occupations; *n* = 379,233.**

| **Variable** | **Parameter Estimate** | **Standard Error** | **Pr > \|*t*\|** |
| --- | --- | --- | --- |
| **Intercept** | 8.04966 | 0.01716 | <.0001 |
| **LEXP** | 0.03727 | 0.00110 | <.0001 |
| **LEXPSQ** | -0.00071050 | 0.00002240 | <.0001 |
| **BLACK** | 0.04080 | 0.00672 | <.0001 |
| **HISP** | -0.01511 | 0.00428 | 0.0004 |
| **GENDER** | 0.16599 | 0.00358 | <.0001 |
| **NOHS** | -0.29400 | 0.01398 | <.0001 |
| **SOMECOL** | 0.00054953 | 0.01202 | 0.9635 |
| **AADEG** | 0.14984 | 0.01725 | <.0001 |
| **BADEG** | 0.36627 | 0.01431 | <.0001 |
| **PROFDEG** | 0.78408 | 0.01420 | <.0001 |
| **GRADDEG** | 0.61170 | 0.00918 | <.0001 |
| **VETSTAT** | -0.06886 | 0.01193 | <.0001 |
| **VETEXP** | -0.00218 | 0.00109 | 0.0454 |
| **VETEXPSQ** | 0.00011746 | 0.00002418 | <.0001 |
| **HWY** | 0.00083566 | 0.00000217 | <.0001 |
| **AGE2** | 0.09322 | 0.01247 | <.0001 |
| **AGE3** | 0.05964 | 0.01591 | 0.0002 |
| **AGE4** | 0.07204 | 0.01764 | <.0001 |
| **AGE5** | 0.05151 | 0.02024 | 0.0109 |
| **A2NOHS** | 0.17227 | 0.01613 | <.0001 |
| **A2ASSOC** | 0.05827 | 0.02014 | 0.0038 |
| **A2SOMCOL** | 0.11946 | 0.01462 | <.0001 |
| **A2BA** | 0.10106 | 0.01656 | <.0001 |
| **A2PGDEG** | 0.13550 | 0.01676 | <.0001 |
| **A3NOHS** | 0.14060 | 0.01596 | <.0001 |
| **A3ASSOC** | 0.07942 | 0.01942 | <.0001 |
| **A3SOMCOL** | 0.10856 | 0.01425 | <.0001 |
| **A3BA** | 0.12133 | 0.01595 | <.0001 |
| **A3PGDEG** | 0.18577 | 0.01343 | <.0001 |
| **A4NOHS** | 0.11591 | 0.01553 | <.0001 |
| **A4ASSOC** | 0.04329 | 0.01871 | 0.0207 |
| **A4SOMCOL** | 0.11146 | 0.01365 | <.0001 |
| **A4BA** | 0.03163 | 0.01528 | 0.0384 |
| **A5NOHS** | 0.25007 | 0.02628 | <.0001 |
| **A5SOMCOL** | 0.13534 | 0.02029 | <.0001 |
| **LAMBDA** | -1.45037 | 0.05431 | <.0001 |
| **F Value** | 10756.6 | <.0001 |  |
| $\bar{R}^{2}$ | 0.5052 |  |  |

Variables’ Definitions are in Table S2.

The same technique was used for the full sample analysis and for each one of the individual occupational analysis. Whenever possible, we estimated each occupation separately.

The previous equation was estimated using the ACS and CPS data. These data sets do not include aptitude tests or AFQT. Since we believe the AFQT value contains substantial information about individual sailors, we used the third set of data (NLS) to estimate a wage equation (using the same method as above) that included the AFQT. This allowed us to capture the AFQT impact on each one of the two equations (selection and wage).

To add the AFQT estimated coefficients from the NLS into our occupation (and generic) estimated coefficient, we need to normalize all of the coefficients such that the mean (first moment) with and without the AFQT is not changed. By adding the AFQT to the equations, the spread of the expected civilian wages and employment probability increases. This result is what we expected.

**The skill groups analyzed (all Navy enlisted personnel)**

Mapping of the skill groups is based on Job families as defined by the Navy. Overall, there are 187 skill groups that were aggregated into 22 groups (called here, ‘Skill Groups’). We thank Navy Personnel Research, Studies and Technology Department (NPRST) for providing us with the necessary mapping information.

- Administration
- Submariner Electronic
- Surface Combat Electronics
- Surface Electrical
- Surface Engineering
- Surface Operations
- Crypto Intel
- Diver Spec War
- Nuclear
- Aviation Mechanical (3 subgroups)
- Aviation Air Crew
- Aviation Boatswain
- Aviation ATC
- Aviation Meteorologist
- Seabee
- Submariner Other
- Surface Combat Weapons
- Surface Repair
- Surface Deck
- Medical (2 subgroups)
- Supply (2 subgroup

# **Additional results of different skill groups**

**Table S4. Bivariate probit model analysis of most Navy skill groups (1997-2008).**

| Skill Group  (Obs.) | ADMIN  (42956) | NUCLEAR (28634) | SCWEAOP  (19000) | SUBELECT (30652) | DRVSPEC  (9236) | AIRCREW (20123) | SURFDECK (33887) | SURFENG (50767) | MEDICAL (125275) |
| --- | --- | --- | --- | --- | --- | --- | --- | --- | --- |
| Stay/Loss Equation | | | | | | | | | |
| BLACK | ***0.579*** | ***0.116*** | ***0.287*** | ***0.306*** | - | ***0.26*** | ***0.298*** | ***0.197*** | *-* |
| HISP | ***0.176*** | *-* | ***0.225*** | - | - | ***0.264*** | ***0.166*** | *-* | ***0.045*** |
| OTHER | ***0.105*** | *-* | *-* | -0.094 | - | ***0.012*** | ***0.155*** | ***0.138*** | ***0.274*** |
| FEMALE | ***0.086*** | ***-0.197*** | ***-0.394*** | NA | -0.741 | ***-0.788*** | ***-0.518*** | ***-0.31*** | *-* |
| HSDIP | - | *-* | -0.089 | - | - | ***-0.373*** | ***-0.137*** | ***-0.113*** | *-* |
| HSPLUS | ***-0.775*** | ***-0.299*** | ***-0.317*** | ***-0.677*** | ***-1.127*** | ***-1.102*** | ***-0.49*** | ***-0.318*** | *-* |
| MARITAL | - | ***0.231*** | ***0.112*** | ***0.238*** | *-* | - | - | ***0.097*** | ***0.13*** |
|  |  |  |  |  |  |  |  |  |  |
| Promotion Equation | | | | | | | | | |
| BLACK | ***-0.043*** | ***-0.213*** | ***-0.078*** | ***-0.065*** | - | - | ***-0.128*** | **-0.149** | ***-0.104*** |
| HISP | - | *-* | - | - | ***-0.158*** | - | ***-0.071*** | - | - |
| OTHER | - | *-* | - | - | - | - | ***-0.145*** | ***-0.064*** | - |
| FEMALE | 0.03 | ***-0.136*** | - | NA | - | - | - | -0.067 | ***0.069*** |
| HSDIP | - | *-* | 0.072 | - | - | - | - | - | - |
| HSPLUS | ***0.107*** | ***0.344*** | 0.154 | - | ***0.244*** | ***0.17*** | - | - | ***0.108*** |
| AFQT | ***0.007*** | ***0.003*** | ***0.005*** | ***0.003*** | ***0.007*** | ***0.005*** | ***0.005*** | ***0.006*** | ***0.005*** |
|  |  |  |  |  |  |  |  |  |  |
|  |  |  |  |  |  |  |  |  |  |
| Correlation | ***0.628*** | ***-0.115*** | **0.707** | **0.181** | 0.162 | ***0.378*** | ***0.429*** | ***0.63*** | ***0.809*** |

The table Presents the Main Coefficients of Interest for Each One of the Two Equations. The Coefficients of Most Interest are the Minority Coefficients of Both Equations (Positive in the Stay/Loss and Negative in Promotion).

1. In the above table italic-bold is significant at least at the 5% level, regular font at least at the 10% level and "-" not significant at the 10%. “NA” is when information is not available.

2. For the following skill groups there were a few statistically significant black-year interactions.-ADMIN, SCWEAOP, SURFDECK, SURFENG, and MEDICAL.

3. For the following skill groups there were a few statistically significant female-year interactions- SURFDECK, SURFENG, MEDICAL and CRYPTO.

4. The SUBELECT skill group had no females in the sample

5. ADMIN and NUCLEAR skill groups estimations are obtained from two combined data sets-(1997-2005) and (2002 – 2008). All other skill groups are from 1997-2005.

| Skill Group (Obs.) | SURFELEC (31490) | SUPPLY (79591) | CRYPTO (36579) | SURFREP (20494) | MECHANIC (165776) | SUBOTHER (20153) | SCELECT (48310) | SURFOPER (69611) | SEABEE (32580) |
| --- | --- | --- | --- | --- | --- | --- | --- | --- | --- |
| Stay/Loss Equation | | | | | | | | | |
| BLACK | - | ***0.214*** | ***0.311*** |  | ***0.039*** |  | ***0.246*** | ***0.187*** | ***0.099*** |
| HISP | - | ***0.208*** | ***0.225*** |  | *-* |  | *-* | ***0.091*** | ***0.207*** |
| OTHER | - | ***0.397*** | ***0.148*** |  | *-* |  | ***-0.106*** | *-* | ***0.208*** |
| FEMALE | ***-1.166*** | ***-0.124*** | ***-0.259*** |  | ***-0.26*** |  | ***-0.699*** | **-0.271** | ***-0.378*** |
| HSDIP | - | ***-0.098*** | ***-*** |  | *-* |  | ***-0.15*** | - | *-* |
| HSPLUS | - | ***-0.334*** | ***-0.404*** |  | ***-0.139*** |  | ***-0.666*** | ***-0.399*** | ***-0.251*** |
| MARITAL | - | ***0.074*** | ***0.17*** |  | ***0.131*** |  | ***0.151*** | ***0.141*** | ***0.083*** |
|  |  |  |  |  |  |  |  |  |  |
| Promotion Equation | | | | | | | | | |
| BLACK | - | ***-0.128*** | ***-0.165*** | ***-0.155*** | ***-0.153*** | ***-0.094*** | ***-0.219*** | ***-0.138*** | ***-0.207*** |
| HISP | - | ***0.081*** | -0.057 | - | ***-0.046*** | ***-0.131*** | - | ***-0.066*** | ***-0.111*** |
| OTHER | - | ***0.04*** | - | - | ***-0.074*** | ***-0.124*** | ***-0.152*** | ***-0.087*** | -0.06 |
| FEMALE | ***-0.172*** | ***0.181*** | - | - | - |  | -0.06 | - | - |
| HSDIP | ***0.145*** | -0.053 | ***0.114*** | - | - | *-* | - | 0.045 | ***0.069*** |
| HSPLUS | ***0.229*** | - | 0.106 | - | - | *-* | - | - | ***0.172*** |
| AFQT | ***0.009*** | ***0.008*** | ***0.004*** | ***0.004*** | ***0.006*** | ***0.004*** | ***0.003*** | ***0.006*** | ***0.006*** |
| Correlation | ***0.999*** | ***0.673*** | ***0.452*** |  | ***0.848*** |  | ***0.742*** | ***0.747*** | ***0.828*** |

**Table S4 (Cont.)**

1. In the above table italic-bold is significant at least at the 5% level, regular font at least at the 10% level and "-" not significant at the 10%. “NA” is when information is not available.
2. For the following skill groups there were a few statistically significant black-year interactions.-, SURFELEC, CRYPTO, SUPPLY, MECHANIC, SCELECT, SURFOPER and SEABEE.
3. For the following skill groups there were a few statistically significant female-year interactions- CRYPTO, MECHANIC, SCELECT and SEABEE.

| **All Pay Grades** | **Race Analysis - Nuclear** | | | | | | | | | | | | | | | |
| --- | --- | --- | --- | --- | --- | --- | --- | --- | --- | --- | --- | --- | --- | --- | --- | --- |
|  | **Bivariate** | | | | | | | | | | | | **Probit** | | | |
|  | **Navy offers promotion - Coefficients** | | | | **Not promoted and stay - Coefficients** | | | | **Promoted and stay - Coefficients** | | | | **Promoted and stay - Coefficients** | | | |
|  | **White** | **Black** | **Hispanic** | **Other** | **White** | **Black** | **Hispanic** | **Other** | **White** | **Black** | **Hispanic** | **Other** | **White** | **Black** | **Hispanic** | **Other** |
| All | 27.9% | 24.4% | 28.4% | 26.2% | 64.9% | 68.7% | 64.3% | 66.8% | 24.2% | 21.3% | 24.4% | 23.1% | 22.1% | 18.5% | 22.4% | 21.1% |
| White | 28.8% | 25.2% | 29.3% | 27.0% | 64.1% | 67.8% | 63.4% | 66.0% | 24.8% | 21.9% | 25.0% | 23.6% | 22.4% | 18.7% | 22.7% | 21.3% |
| Black | 27.2% | 23.7% | 27.6% | 25.5% | 65.1% | 68.9% | 64.5% | 67.1% | 23.7% | 20.8% | 23.8% | 22.5% | 22.3% | 18.6% | 22.5% | 21.3% |
| Hispanic | 24.5% | 21.2% | 24.8% | 23.4% | 68.3% | 71.9% | 67.8% | 69.8% | 22.0% | 19.2% | 22.1% | 21.2% | 21.0% | 17.4% | 21.0% | 20.2% |
| Other | 21.5% | 18.6% | 21.6% | 20.7% | 71.9% | 75.2% | 71.6% | 73.2% | 19.9% | 17.2% | 19.8% | 19.2% | 19.7% | 16.1% | 19.5% | 18.9% |
| **E4** | **Bivariate** | | | | | | | | | | | | **Probit** | | | |
| All | 67.4% | 61.3% | 63.1% | 65.2% | 31.9% | 37.8% | 36.1% | 34.1% | 66.1% | 59.9% | 61.6% | 63.9% | 67.6% | 58.1% | 62.5% | 64.5% |
| White | 68.0% | 61.9% | 63.7% | 65.8% | 31.4% | 37.2% | 35.5% | 33.5% | 66.7% | 60.5% | 62.2% | 64.5% | 68.0% | 58.6% | 62.9% | 64.9% |
| Black | 64.0% | 57.6% | 59.4% | 61.7% | 35.3% | 41.4% | 39.6% | 37.5% | 62.5% | 56.0% | 57.8% | 60.2% | 65.3% | 55.5% | 60.1% | 62.1% |
| Hispanic | 64.9% | 58.6% | 60.4% | 62.6% | 34.5% | 40.5% | 38.7% | 36.6% | 63.6% | 57.3% | 59.0% | 61.3% | 65.5% | 56.0% | 60.4% | 62.3% |
| Other | 65.5% | 59.1% | 61.0% | 63.2% | 33.8% | 40.0% | 38.1% | 36.0% | 64.2% | 57.7% | 59.5% | 61.9% | 66.7% | 56.9% | 61.4% | 63.4% |
| **E6** | **Bivariate** | | | | | | | | | | | | **Probit** | | | |
| All | 22.9% | 19.3% | 24.6% | 17.8% | 64.1% | 69.1% | 62.4% | 69.5% | 16.0% | 14.0% | 17.0% | 12.6% | 9.1% | 8.3% | 10.8% | 7.6% |
| White | 24.3% | 20.5% | 25.9% | 19.0% | 62.9% | 67.9% | 61.2% | 68.4% | 16.8% | 14.8% | 17.9% | 13.3% | 9.6% | 8.8% | 11.4% | 8.1% |
| Black | 20.7% | 17.2% | 22.3% | 15.7% | 65.2% | 70.4% | 63.3% | 70.7% | 14.8% | 12.7% | 15.8% | 11.4% | 8.1% | 7.4% | 9.7% | 6.7% |
| Hispanic | 14.6% | 11.8% | 16.0% | 10.6% | 71.2% | 75.9% | 69.5% | 76.0% | 10.6% | 8.8% | 11.5% | 7.8% | 5.7% | 5.2% | 7.0% | 4.7% |
| Other | 12.1% | 9.7% | 13.3% | 8.7% | 75.3% | 79.8% | 73.7% | 79.9% | 9.4% | 7.7% | 10.2% | 6.8% | 4.5% | 4.0% | 5.5% | 3.6% |

**Further results of the highly qualified nuclear skill group**

**Table S5. Detailed analyses by race for the highly skilled nuclear skill group (1997 – 2008).**

**Comparison of administration, cryptology nuclear groups**

**Table S6. Descriptive statistics for three representative Navy skill groups.**

| **Everyone** | All | White | Non-White | Black | Male | Female |
| --- | --- | --- | --- | --- | --- | --- |
| AFQT | 55.0 | 63.0 | 48.9 | 46.5 | 56.3 | 52.0 |
| Months in Rank | 29.8 | 31.2 | 28.6 | 29.4 | 31.0 | 26.8 |
| Performance Mark Av. (PMA) | 3.1 | 3.1 | 3.2 | 3.2 | 3.1 | 3.2 |
| Months at Sea | 41.6 | 40.81 | 42.13 | 42.28 | 44.63 | 34.37 |
| Unemp. Prob (%) | 6 | 4 | 7 | 8 | 5 | 7 |
| High School Diploma (%) | 88 | 87 | 89 | 92 | 87 | 91 |
| > High School (%) | 7 | 7 | 6 | 5 | 7 | 6 |
| Multiple Exams (%) | 32 | 32 | 33 | 34 | 33 | 31 |
| **Selected for Promotion** |  |  |  |  |  |  |
| AFQT | 56.6 | 64.3 | 49.7 | 47.3 | 57.8 | 53.7 |
| Months in Rank | 41.3 | 42.1 | 40.6 | 42.2 | 42.7 | 38.1 |
| PMA | 3.2 | 3.2 | 3.3 | 3.3 | 3.2 | 3.3 |
| Months at Sea | 36.67 | 36.37 | 36.94 | 36.78 | 39.74 | 29.46 |
| Unemp. Prob (%) | 6 | 4 | 7 | 8 | 5 | 7 |
| High School Diploma (%) | 87 | 86 | 88 | 91 | 86 | 90 |
| > High School (%) | 7 | 7 | 7 | 5 | 8 | 7 |
| Multiple Exams (%) | 17 | 15 | 19 | 19 | 17 | 17 |

**Nuclear**

| **Everyone** | **All** | **White** | **Non-White** | **Black** | **Male** | **Female** |
| --- | --- | --- | --- | --- | --- | --- |
| AFQT | 88.7 | 88.89 | 87.79 | 86.7 | 88.69 | 89.12 |
| Months in Rank | 26.1 | 26.81 | 22.75 | 26.94 | 26.37 | 17.6 |
| PMA | 3.46 | 3.45 | 3.47 | 3.47 | 3.46 | 3.38 |
| Months at Sea | 39.93 | 40.51 | 37.18 | 41.33 | 40.31 | 28.1 |
| Unemp. Prob (%) | 5 | 4 | 6 | 9 | 5 | 7 |
| High School Diploma (%) | 97 | 97 | 97 | 95 | 97 | 96 |
| > High School (%) | 3 | 3 | 3 | 4 | 3 | 4 |
| Multiple Exams (%) | 12 | 13 | 11 | 14 | 12 | 11 |
| **Selected for Promotion** |  |  |  |  |  |  |
| AFQT | 88.57 | 88.79 | 87.28 | 85.93 | 88.56 | 88.84 |
| Months in Rank | 41.48 | 42.45 | 35.71 | 43.53 | 41.94 | 22.59 |
| PMA | 3.66 | 3.65 | 3.68 | 3.77 | 3.66 | 3.72 |
| Months at Sea | 42.26 | 42.82 | 38.89 | 43.72 | 42.66 | 25.93 |
| Unemp. Prob. (%) | 4 | 4 | 6 | 8 | 4 | 7 |
| High School Diploma (%) | 96 | 96 | 96 | 92 | 96 | 96 |
| > High School (%) | 4 | 4 | 4 | 7 | 4 | 4 |
| Multiple Exams (%) | 9 | 9 | 10 | 8 | 9 | 13 |

| **Cryptology Full Sample** | All | White | Non White | Blacks | Male | Female |
| --- | --- | --- | --- | --- | --- | --- |
| AFQT | 68.7 | 72.5 | 60.5 | 57.1 | 69.9 | 65.4 |
| Time in Rank | 29.9 | 30.6 | 28.4 | 29.9 | 30.5 | 28.2 |
| PMA | 3.4 | 3.4 | 3.5 | 3.5 | 3.4 | 3.5 |
| Unemp. Prob | 0.44 | 0.41 | 0.51 | 0.54 | 0.42 | 0.48 |
| Months at Sea | 30.8 | 31.1 | 30.4 | 30.4 | 35.1 | 18.3 |
| HS (%) | 0.89 | 0.89 | 0.90 | 0.91 | 0.89 | 0.90 |
| MTHS (%) | 0.07 | 0.07 | 0.06 | 0.05 | 0.06 | 0.08 |
| Took Exam more than once (%) | 0.29 | 0.28 | 0.32 | 0.33 | 0.28 | 0.32 |
| **Cryptology Selected for Promotion** |  |  |  |  |  |  |
| AFQT | 69.3 | 72.7 | 61.0 | 57.1 | 70.3 | 66.3 |
| Time in Rank | 35.0 | 35.8 | 33.0 | 35.6 | 35.9 | 32.2 |
| PMA | 3.5 | 3.4 | 3.5 | 3.6 | 3.4 | 3.5 |
| Unemp. Prob | 0.44 | 0.41 | 0.51 | 0.55 | 0.43 | 0.48 |
| Months at Sea | 28.1 | 28.4 | 27.5 | 27.7 | 32.0 | 16.5 |
| HS (%) | 0.89 | 0.89 | 0.9 | 0.92 | 0.89 | 0.90 |
| MTHS (%) | 0.07 | 0.07 | 0.05 | 0.05 | 0.08 | 0.06 |
| **Took Exam more than once (%)** | 0.13 | 0.12 | 0.16 | 0.16 | 0.13 | 0.16 |

*Notes*: AFQT is the department of defense IQ test. The PMA is the Performance Mark Average: Early Promote (4.0 points), Must Promote (3.8), Promotable (3.6), Progressing (3.4), and Significant Problems (2.0 points). “Multiple exams” means that the sailor took the exam more than once for the most recent promotion opportunity.

**A detailed comparison of administration and nuclear groups**

**Table S7. A detailed race and gender decomposition comparisons of the administration and nuclear skill groups (1997 – 2008).**

| **Race – All Pay Grades** | **Promotion Probabilities** | | **Stay and Not promoted Probabilities** | |
| --- | --- | --- | --- | --- |
|  | **Admin** | **Nuclear** | **Admin** | **Nuclear** |
| White coeff * white char | ***33.7*** | ***28.8*** | ***61.0*** | ***64.1*** |
| White coeff & black char | 32.4 | 25.2 | 64.6 | 67.9 |
| White coeff & Hispanic char | 33.5 | 29.3 | 62.3 | 63.4 |
| White coeff & Other char | 33.7 | 27.0 | 61.8 | 66.0 |
| Black coeff & white char | 29.5 | 27.2 | 63.4 | 65.1 |
| Black coeff & black char | ***28.8*** | ***23.7*** | ***67.5*** | ***69.0*** |
| Black coeff & Hispanic char | 30.1 | 27.6 | 64.2 | 64.5 |
| Black coeff & Other char | 29.5 | 25.5 | 64.4 | 67.1 |
| Hispanic coeff & White char | 28.6 | 24.5 | 66.2 | 68.3 |
| Hispanic coeff & Black char | 27.3 | 21.2 | 70.1 | 71.9 |
| Hispanic coeff & Hispanic char | ***28.5*** | ***24.8*** | ***67.4*** | ***68.0*** |
| Hispanic coeff & Other char | 28.5 | 23.4 | 67.0 | 69.8 |
| Other coeff & White char | 29.0 | 21.5 | 66.5 | 71.9 |
| Other coeff & Black char | 28.1 | 18.6 | 69.7 | 75.2 |
| Other coeff & Hispanic char | 29.2 | 21.6 | 67.2 | 71.6 |
| Other coeff & Other char | ***29.0*** | ***20.7*** | ***67.2*** | ***72.8*** |
| **Gender – All Pay Grades** |  | | | |
| Male coeff & male char | ***31.0*** | ***27.9*** | ***64.7*** | ***65.0*** |
| Male coeff & female char | 28.6 | 25.6 | 67.4 | 65.2 |
| Female coeff & male char | 30.0 | 24.0 | 64.7 | 70.3 |
| Female coeff & female char | ***30.5*** | ***22.0*** | ***64.7*** | ***70.3*** |

**Table S7 (Cont.).**

| **Race – Pay Grade E4** | **Promotion Probabilities** | | **Stay and Not promoted Probabilities** | |
| --- | --- | --- | --- | --- |
|  | **Admin** | **Nuclear** | **Admin** | **Nuclear** |
| White coeff & white char | ***40.7%*** | ***68.0%*** | ***56.6%*** | ***31.4%*** |
| White coeff & black char | 34.8% | 61.9% | 63.7% | 37.2% |
| White coeff & Hispanic char | 36.9% | 63.7% | 60.6% | 35.5% |
| White coeff & Other char | 40.7% | 65.8% | 56.2% | 33.5% |
| Black coeff & white char | 39.0% | 64.0% | 57.0% | 35.3% |
| Black coeff & black char | ***34.2%*** | ***57.6%*** | ***63.5%*** | ***41.4%*** |
| Black coeff & Hispanic char | 36.4% | 59.4% | 59.8% | 39.6% |
| Black coeff & Other char | 39.0% | 61.7% | 56.5% | 37.5% |
| Hispanic coeff & White char | 36.6% | 64.9% | 59.9% | 34.5% |
| Hispanic coeff & Black char | 31.6% | 58.6% | 66.5% | 40.5% |
| Hispanic coeff & Hispanic char | ***33.7%*** | ***60.4%*** | ***63.0%*** | ***38.7%*** |
| Hispanic coeff & Other char | 36.6% | 62.6% | 59.4% | 36.6% |
| Other coeff & White char | 36.0% | 65.5% | 60.5% | 33.8% |
| Other coeff & Black char | 31.0% | 59.1% | 67.0% | 40.0% |
| Other coeff & Hispanic char | 33.1% | 61.0% | 63.8% | 38.1% |
| Other coeff & Other char | ***36.0%*** | ***63.2%*** | ***60.1%*** | ***36.0%*** |
| **Gender – Pay Grade E4** |  | | | |
| Male coeff & male char | ***38.5%*** | NA | **58.8%** | NA |
| Male coeff & female char | 35.5% | NA | 62.1% | NA |
| Female coeff & male char | 33.4% | NA | 63.2% | NA |
| Female coeff & female char | **33.9%** | NA | **63.2%** | NA |

**Table S7 (Cont.).**

| **Race – Pay Grade E6** | **Promotion Probabilities** | | **Stay and Not promoted Probabilities** | |
| --- | --- | --- | --- | --- |
|  | **Admin** | **Nuclear** | **Admin** | **Nuclear** |
| White coeff & white char | ***27.3%*** | ***24.3%*** | ***65.0%*** | ***62.9%*** |
| White coeff & black char | 27.3% | 20.5% | 68.2% | 67.9% |
| White coeff & Hispanic char | 28.1% | 25.9% | 66.2% | 61.2% |
| White coeff & Other char | 27.3% | 19.0% | 66.4% | 68.4% |
| Black coeff & white char | 19.4% | 20.7% | 69.5% | 65.2% |
| Black coeff & black char | ***20.0%*** | ***17.2%*** | ***74.1%*** | **70.4%** |
| Black coeff & Hispanic char | 20.9% | 22.3% | 71.3% | 63.3% |
| Black coeff & Other char | 19.4% | 15.7% | 71.8% | 70.7% |
| Hispanic coeff & White char | 17.5% | 14.6% | 74.1% | 71.2% |
| Hispanic coeff & Black char | 17.8% | 11.8% | 78.0% | 75.9% |
| Hispanic coeff & Hispanic char | ***18.6%*** | ***16.0%*** | ***75.7%*** | **69.5%** |
| Hispanic coeff & Other char | 17.5% | 10.6% | 77.0% | 76.0% |
| Other coeff & White char | 23.0% | 12.1% | 71.2% | 75.3% |
| Other coeff & Black char | 23.3% | 9.7% | 73.7% | 74.3% |
| Other coeff & Hispanic char | 24.1% | 13.3% | 71.8% | 80.0% |
| Other coeff & Other char | ***23.0%*** | ***8.7%*** | ***72.4%*** | ***79.4%*** |
| **Gender– Pay Grade E6** |  | | | |
| Male coeff & male char | ***23.2%*** | NA | ***70.8%*** | NA |
| Male coeff & female char | 21.0% | NA | 73.3% | NA |
| Female coeff & male char | 24.1% | NA | 66.8% | NA |
| Female coeff & female char | ***24.8%*** | NA | **66.9%** | NA |

**Table S8. Peace vs. war periods analyses by gender for the highly skilled nuclear skill group.**

| **All** | **Gender Analyses – Peace Time - Nuclear** | | | | | | | |
| --- | --- | --- | --- | --- | --- | --- | --- | --- |
|  | **Bivariate** | | | | | | **Probit** | |
|  | **Navy offers promotion - Coefficients** | | **Not promoted and stay - Coefficients** | | **Promoted and stay - Coefficients** | | **Promoted and stay - Coefficients** | |
|  | **Male** | **Female** | **Male** | **Female** | **Male** | **Female** | **Male** | **Female** |
| All | 53.3% | 50.6% | 41.4% | 46.2% | 44.2% | 45.8% | 39.0% | 34.0% |
| Male | 53.2% | 50.5% | 41.4% | 46.2% | 44.0% | 45.6% | 38.6% | 33.6% |
| Female | 58.5% | 55.8% | 41.3% | 44.2% | 58.0% | 55.8% | 61.5% | 55.5% |
| **All** | **Gender Analyses – War Time - Nuclear** | | | | | | | |
|  | **Bivariate** | | | | | | **Probit** | |
|  | **Navy offers promotion - Coefficients** | | **Not promoted and stay - Coefficients** | | **Promoted and stay - Coefficients** | | **Promoted and stay - Coefficients** | |
|  | **Male** | **Female** | **Male** | **Female** | **Male** | **Female** | **Male** | **Female** |
| All | 15.0% | 13.6% | 77.3% | 74.5% | 13.9% | 12.1% | 13.3% | 11.9% |
| Male | 14.9% | 13.5% | 77.4% | 74.6% | 13.8% | 12.0% | 13.2% | 11.8% |
| Female | 16.3% | 14.8% | 77.8% | 75.6% | 15.7% | 13.9% | 15.9% | 14.4% |

**Table S9. A more refined peace vs. war periods analyses by race for the nuclear skill group.**

| **All** | **Race Analyses – Peace Time - Nuclear** | | | | | | | | | | | | | | | |
| --- | --- | --- | --- | --- | --- | --- | --- | --- | --- | --- | --- | --- | --- | --- | --- | --- |
|  | **Bivariate** | | | | | | | | | | | | **Probit** | | | |
|  | **Navy offers promotion - Coefficients** | | | | **Not promoted and stay - Coefficients** | | | | **Promoted and stay - Coefficients** | | | | **Promoted and stay - Coefficients** | | | |
|  | **White** | **Black** | **Hispanic** | **Other** | **White** | **Black** | **Hispanic** | **Other** | **White** | **Black** | **Hispanic** | **Other** | **White** | **Black** | **Hispanic** | **Other** |
| All | 53.3% | 49.1% | 55.3% | 52.1% | 41.3% | 45.1% | 39.7% | 42.9% | 44.2% | 41.2% | 45.7% | 44.1% | 38.6% | 36.4% | 39.9% | 37.2% |
| White | 53.3% | 49.1% | 55.3% | 52.1% | 41.2% | 45.0% | 39.6% | 42.8% | 43.9% | 41.0% | 45.5% | 43.8% | 38.3% | 35.8% | 39.2% | 36.6% |
| Black | 55.7% | 51.3% | 57.6% | 54.4% | 38.2% | 42.4% | 36.6% | 40.1% | 44.0% | 42.2% | 45.6% | 45.0% | 42.6% | 39.9% | 43.6% | 40.7% |
| Hispanic | 50.2% | 46.0% | 52.1% | 49.0% | 45.0% | 48.7% | 43.4% | 46.3% | 44.0% | 40.8% | 45.7% | 43.4% | 40.8% | 38.2% | 41.7% | 39.0% |
| Other | 55.4% | 51.2% | 57.3% | 54.1% | 41.8% | 45.8% | 40.0% | 43.1% | 50.8% | 47.3% | 52.5% | 50.2% | 49.2% | 46.5% | 50.2% | 47.4% |
| Male | 53.3% | 49.1% | 55.2% | 52.0% | 41.3% | 45.1% | 39.7% | 42.9% | 44.0% | 41.1% | 45.6% | 43.9% | 38.7% | 36.2% | 39.6% | 37.0% |
| Female | 56.0% | 51.6% | 57.9% | 54.7% | 44.0% | 48.4% | 42.0% | 45.3% | 55.8% | 51.6% | 57.8% | 54.7% | 55.8% | 52.5% | 56.9% | 53.6% |
| **All** | **Gender Analyses – War Time - Nuclear** | | | | | | | | | | | | | | | |
|  | **Bivariate** | | | | | | | | | | | | **Probit** | | | |
|  | **Navy offers promotion - Coefficients** | | | | **Not promoted and stay - Coefficients** | | | | **Promoted and stay - Coefficients** | | | | **Promoted and stay - Coefficients** | | | |
|  | **White** | **Black** | **Hispanic** | **Other** | **White** | **Black** | **Hispanic** | **Other** | **White** | **Black** | **Hispanic** | **Other** | **White** | **Black** | **Hispanic** | **Other** |
| All | 15.3% | 11.0% | 14.8% | 13.7% | 77.0% | 79.1% | 77.8% | 78.2% | 14.2% | 10.0% | 13.7% | 12.6% | 13.5% | 9.9% | 13.3% | 13.0% |
| White | 15.3% | 11.0% | 16.2% | 13.7% | 76.9% | 79.0% | 77.7% | 78.1% | 14.1% | 9.9% | 15.3% | 12.6% | 13.5% | 9.9% | 13.3% | 13.0% |
| Black | 16.7% | 12.2% | 14.8% | 15.0% | 76.6% | 79.2% | 77.4% | 77.9% | 15.7% | 11.3% | 13.7% | 14.1% | 14.3% | 10.5% | 14.1% | 13.7% |
| Hispanic | 14.8% | 10.7% | 14.3% | 13.3% | 77.8% | 79.9% | 78.6% | 79.0% | 13.9% | 9.9% | 13.5% | 12.4% | 13.4% | 9.9% | 13.2% | 12.9% |
| Other | 14.9% | 10.7% | 14.4% | 13.3% | 77.6% | 79.7% | 78.4% | 78.8% | 14.1% | 10.0% | 13.6% | 12.6% | 13.3% | 9.8% | 13.1% | 12.8% |
| Male | 15.3% | 11.0% | 14.8% | 13.7% | 77.2% | 79.3% | 78.0% | 78.4% | 14.2% | 10.0% | 13.8% | 12.6% | 13.4% | 9.9% | 13.2% | 12.9% |
| Female | 15.4% | 11.1% | 14.9% | 13.8% | 75.2% | 77.0% | 76.1% | 76.4% | 14.4% | 10.2% | 14.0% | 12.9% | 14.7% | 11.0% | 14.5% | 14.2% |

**Race and gender differential - further tests**

**Table S10. Predicted probabilities by race and sex for the nuclear skill group.**

| **Promotion Probabilities by Pay Grade and Race - Nuclear -Overall** | | | | | | | | | |  |  |
| --- | --- | --- | --- | --- | --- | --- | --- | --- | --- | --- | --- |
|  | | Black | | White | | Hispanic | | Other | |  |  |
| Black | | ***0.237*** | | 0.272 | | 0.240 | | 0.222 | |  |  |
| White | | 0.252 | | ***0.288*** | | 0.293 | | 0.270 | |  |  |
| Hispanic | | 0.213 | | 0.245 | | ***0.248*** | | 0.236 | |  |  |
| Other | | 0.178 | | 0.290 | | 0.186 | | ***0.207*** | |  |  |
| **E4 -E5** | | | | | | | | | |  |  |
|  | | Black | | White | | Hispanic | | Other | |  |  |
| Black | | ***0.576*** | | 0.640 | | 0.528 | | 0.551 | |  |  |
| White | | 0.619 | | ***0.680*** | | 0.637 | | 0.658 | |  |  |
| Hispanic | | 0.540 | | 0.649 | | ***0.604*** | | 0.581 | |  |  |
| Other | | 0.567 | | 0.360 | | 0.586 | | ***0.632*** | |  |  |
| **E6 -E7** | | | | | | | | | |  |  |
|  | | Black | | White | | Hispanic | | Other | |  |  |
| Black | | ***0.172*** | | 0.207 | | 0.186 | | 0.128 | |  |  |
| White | | 0.205 | | ***0.243*** | | 0.259 | | 0.190 | |  |  |
| Hispanic | | 0.129 | | 0.146 | | ***0.160*** | | 0.117 | |  |  |
| Other | | 0.068 | | 0.230 | | 0.073 | | ***0.087*** | |  |  |
| **Stay and Not promoted Probabilities by Pay Grade and Race - Nuclear -Overall** | | | | | | | | | |  |  |
|  | | Black | | White | | Hispanic | | Other | |  |  |
| Black | | ***0.690*** | | 0.651 | | 0.687 | | 0.704 | |  |  |
| White | | 0.679 | | ***0.641*** | | 0.635 | | 0.656 | |  |  |
| Hispanic | | 0.717 | | 0.683 | | ***0.680*** | | 0.690 | |  |  |
| Other | | 0.680 | | 0.665 | | 0.742 | | ***0.727*** | |  |  |
| **E4 -E5** | | | | | | | | | |  |  |
|  | | Black | | White | | Hispanic | | Other | |  |  |
| Black | | ***0.414*** | | 0.353 | | 0.459 | | 0.436 | |  |  |
| White | | 0.373 | | ***0.314*** | | 0.355 | | 0.334 | |  |  |
| Hispanic | | 0.448 | | 0.345 | | ***0.387*** | | 0.408 | |  |  |
| Other | | 0.668 | | 0.387 | | 0.404 | | ***0.360*** | |  |  |
| **E6 -E7** | | | | | | | | | |  |  |
|  | | Black | | White | | Hispanic | | Other | |  |  |
| Black | | ***0.706*** | | 0.652 | | 0.693 | | 0.752 | |  |  |
| White | | 0.680 | | ***0.629*** | | 0.615 | | 0.679 | |  |  |
| Hispanic | | 0.749 | | 0.712 | | ***0.699*** | | 0.745 | |  |  |
| Other | | 0.743 | | 0.699 | | 0.800 | | ***0.794*** | |  |  |
| **Promotion Probabilities by Pay Grade and Gender - Nuclear -Overall** | | | | | | **Stay and Not promoted Probabilities by Pay Grade and Gender - Nuclear -Overall** | | | | | |
|  |  |  |  |  |  |  |  |  |  |  |  |
|  | | Female | | Male | |  | | Female | | Male | |
| Female | | ***0.22*** | | 0.24 | | Female | | ***0.703*** | | 0.703 | |
|  | |  | |  | |  | |  | |  | |
| Male | | 0.256 | | ***0.279*** | | Male | | 0.652 | | ***0.65*** | |

**Additional simulated result: simulated experiments based on the nuclear skill group characteristics and bivariate estimates**

| **Promotion Equation** | |
| --- | --- |
| Months of Service (LOS) | 78.301 |
| Time in Rank (TIR) | 26.105 |
| Demand (Vacants/Takers) | 0.343 |
| AFQT | 85 |
| PMA | 3. 457 |
| PNA | 2. 902 |
| Cycle Cut | 156.389 |
| Indiv. Score | 138.614 |
| Months at Sea/Months of Service | 0.505 |
| **Stay and Loss Equation** |  |
| Base Pay | 26878.100 |
| Expected Civ. Wage | 17006.800 |
| Unemployment | 5.007 |
| Lag Annual GDP | 10034.000 |

**Table S11A. Basic personal, Navy and civilian characteristics of a base case individual (at means of nuclear skill group and at E4).**

**Table S11B. Simulated scenarios for the nuclear skill group.**

| Nuclear | | White-Male | Black-Male | Hispanic-Male |
| --- | --- | --- | --- | --- |
|  |  | Navy offers promotion | Navy offers promotion | Navy offers promotion |
|  | Base Case | 93.9% | 91.0% | 91.9% |
| Personal | AFQT=95 | 94.3% | 91.5% | 92.4% |
|  | AFQT=55 | 92.6% | 89.2% | 90.3% |
|  | HSPLUS | 95.8% | 93.5% | 94.2% |
|  | Not Married | 93.9% | 91.0% | 91.9% |
|  | PMA=3.8 | 97.4% | 95.9% | 96.4% |
|  | PMA=3 | 84.7% | 79.2% | 80.9% |
|  | E5=1 | 17.5% | 12.7% | 20.5% |
| Navy | SS_Sea = 0 | 93.4% | 90.3% | 94.7% |
|  | Sea_Loss+50% | 92.5% | 89.0% | 93.9% |
|  | Demand+50% | 92.2% | 88.7% | 93.7% |
|  | Demand-50% | 91.4% | 87.7% | 93.0% |
|  | Transfer=1 | 71.0% | 63.5% | 74.7% |
|  | | Not promote-Stay | Not promote-Stay | Not promote-Stay |
|  | Base Case | 5.9% | 8.7% | 7.8% |
| Personal | AFQT=95 | 5.5% | 8.1% | 7.3% |
|  | AFQT=55 | 7.2% | 10.4% | 9.3% |
|  | HSPLUS | 4.1% | 6.2% | 5.5% |
|  | Not Married | 5.7% | 8.4% | 7.6% |
|  | PMA=3.8 | 2.5% | 4.0% | 3.5% |
|  | PMA=3 | 14.6% | 19.6% | 18.0% |
|  | E5=1 | 76.9% | 80.6% | 74.0% |
| Navy | SS_Sea = 0 | 6.4% | 9.3% | 5.1% |
|  | Sea_Loss+50% | 7.4% | 10.7% | 6.0% |
|  | Demand+50% | 7.5% | 10.8% | 6.1% |
|  | Demand-50% | 8.3% | 11.8% | 6.7% |
|  | Transfer=1 | 27.0% | 33.5% | 23.6% |
|  | | Promote Stay | Promote Stay | Promote Stay |
|  | Base Case | 88.7% | 85.6% | 86.3% |
| Personal | AFQT=95 | 89.1% | 86.1% | 86.8% |
|  | AFQT=55 | 87.3% | 83.8% | 84.7% |
|  | HSPLUS | 88.4% | 85.9% | 86.4% |
|  | Not Married | 85.5% | 82.4% | 83.0% |
|  | PMA=3.8 | 92.1% | 90.4% | 90.7% |
|  | PMA=3 | 79.5% | 73.9% | 75.4% |
|  | E5=1 | 16.5% | 11.7% | 19.3% |
| Navy | SS_Sea = 0 | 88.3% | 85.0% | 89.3% |
|  | Sea_Loss+50% | 89.1% | 85.6% | 90.3% |
|  | Demand+50% | 87.0% | 83.3% | 88.1% |
|  | Demand-50% | 86.2% | 82.3% | 87.4% |
|  | Transfer=1 | 66.0% | 58.5% | 69.3% |

**Cryptology skill group example**

**Table S12. Promotion probabilities by pay grade, race and sex for the cryptology skill group (Years: 1997-2005).**

|  | **All** | **White** | **Black** | **Hispanic** | **Other** | **Non white** | **Male** | **Female** |
| --- | --- | --- | --- | --- | --- | --- | --- | --- |
| **Overall** | 0.492 | 0.506 | 0.441 | 0.48 | 0.52 | 0.46 | 0.492 | 0.491 |
| **E3 to E4** | 0.912 | 0.921 | 0.888 | 0.9 | 0.887 | 0.894 | 0.911 | 0.915 |
| **E4 to E5** | 0.734 | 0.759 | 0.659 | 0.722 | 0.733 | 0.686 | 0.756 | 0.688 |
| **E5 to E6** | 0.416 | 0.439 | 0.36 | 0.375 | 0.352 | 0.368 | 0.428 | 0.38 |
| **E6 to E7** | 0.294 | 0.316 | 0.235 | 0.221 | 0.262 | 0.234 | 0.301 | 0.264 |
