# Supplementary figures and images for: Does the U.S. Navy’s reliance on objective standards prevent discrimination in promotions and retentions?
Source: PLoS One. 2021 Apr 28;16(4):e0250630. doi: 10.1371/journal.pone.0250630 (PMC8081226; doi:10.1371/journal.pone.0250630)

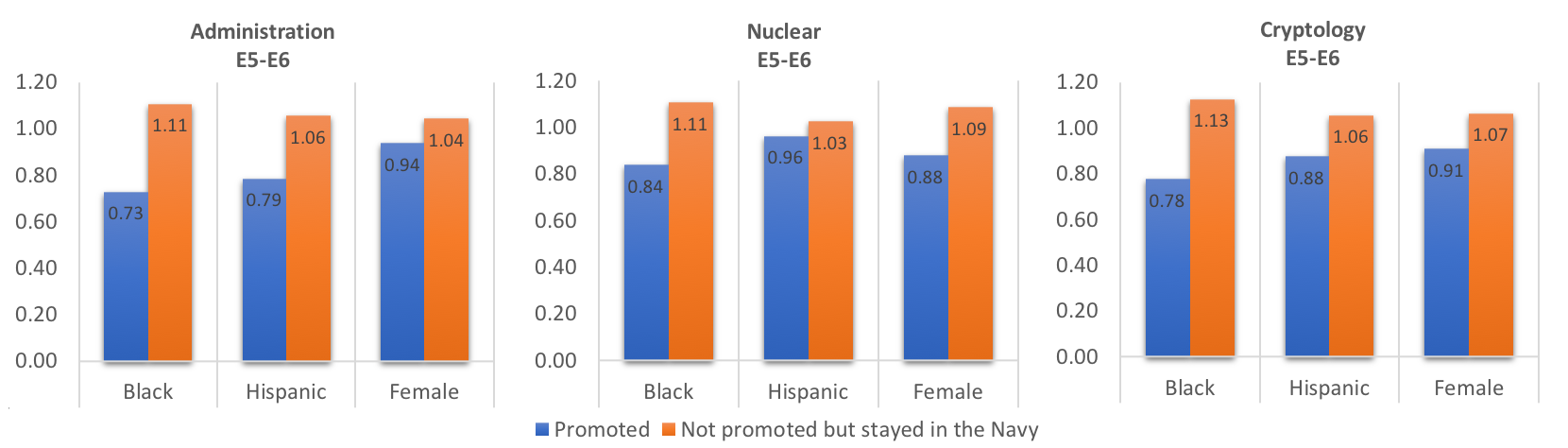

Supplement: S1 Fig — (TIF) [file pone.0250630.s002.tif]
